# Comprehensive Analysis of Regulatory Factors and Immune-Associated Patterns to Decipher Common and *BRCA1/2* Mutation-Type-Specific Critical Regulation in Breast Cancer

**DOI:** 10.3389/fcell.2021.750897

**Published:** 2021-10-18

**Authors:** Yue Li, Wei Dong, Pengqian Zhang, Ting Zhang, Ling Ma, Meng Qu, Xingcong Ma, Xiaoyan Zhou, Qian He

**Affiliations:** ^1^Department of Clinical Laboratory, The Second Affiliated Hospital of Xi’an Jiaotong University, Xi’an, China; ^2^Department of Oncology, The Second Affiliated Hospital of Xi’an Jiaotong University, Xi’an, China

**Keywords:** breast cancer, *BRCA1/2* mutations, weighted gene co-expression network analysis (WGCNA), consensus WGCNA, tumor immune microenvironment

## Abstract

**Background:** *BRCA*1/2 mutations are closely related to high lifetime risk of breast cancer (BC). The objective of this study was to identify the genes, regulators, and immune-associated patterns underlying disease pathology in BC with *BRCA1/2* somatic mutations and their associations with clinical traits.

**Methods:** RNA sequencing data and clinical information from The Cancer Genome Atlas (TCGA; *N* = 36 *BRCA1*-mutant BC; *N* = 49 *BRCA2*-mutant BC; and *N* = 117 *BRCA1/2*-wild-type BC samples) were used for discovery, which included consensus network analysis, function enrichment, and analysis of hub genes; other TCGA data (*N* = 117 triple-negative BC) and two Gene Expression Omnibus database expression profiles were used as validation cohorts.

**Results:** Consensus network analysis helped to identify specific co-expressed modules that showed positive correlations with tumor stage, number of positive lymph nodes, and margin status in *BRCA1/2*-mutant BC but lacking correlations in *BRCA1/2*-wild-type BC. Functional enrichment suggested potential mechanisms in *BRCA1/2* carriers that could regulate the cell cycle, immune response, cellular metabolic processes, and cell migration, via enriched pathways including p53 and JAK–STAT signaling. Consensus network analysis identified the specific and common carcinogenic mechanisms involving *BRCA* mutations. Regulators cross-linking these modules include E2F or IRF transcription factor family, associated with cell cycle or immune response regulation module, respectively. Eight hub genes, including *ISG15*, *BUB1*, and *TTK*, were upregulated in several *BRCA1/2*-mutant BC datasets and showed prognostic value in BC. Furthermore, their genetic expression was related to higher levels of immune infiltration in *BRCA1/2*-mutant BC, which manifested as recruitment of T helper cells (Th1 cells), follicular helper T cells, and regulatory T cells, and T cell exhaustion. Moreover, important indicators for evaluation of BC immunotherapy, tumor mutational burden and neoantigen load also positively correlated with expression of some hub genes.

**Conclusion:** We constructed a *BRCA1/2* mutation-type-specific co-expressed gene network with related transcription factors and immune-associated patterns that could regulate and influence tumor metastasis and immune microenvironment, providing novel insights into the pathological process of this disease and the corresponding *BRCA* mutations.

## Introduction

Breast cancer (BC) is highly heterogeneous with potentially aggressive and complex biological features. Gene expression profiling has been used to analyze this complexity and provide new insights (Holm et al., 2012; Jonsson et al., 2012). Certain genetic mutations typically lead to distinct subtypes of tumors. Sporadic basal-like cancer has phenotypically similarity and relationships with *BRCA* (BC susceptibility gene)-associated BC (Richardson et al., 2006; Alimonti et al., 2010). *BRCA1* and *BRCA2* are tumor suppressor genes with critical roles in DNA damage repair; their germline or somatic mutations are closely related to carcinogenesis, including BC and high-grade serous cancer of the gynecological tract (particularly the fallopian tube, ovary, and peritoneum) (Foulkes and Shuen, 2013). *BRCA1/2* mutations are the most common germline mutations and confer substantial lifetime risk of tumors, accounting for up to 40% of familial BC cases (Anastasiadi et al., 2017). Vidula et al. (2020) evaluated the characteristics of somatically acquired *BRCA1/2* mutations in BC patients and found that 13.5% had somatic *BRCA1/2* mutations (of which 4% were known germline pathogenic variants and the remainder novel variants) in metastatic BC. Germline variants of *BRCA1/2* mutations in BC have been well described, whereas the influence of somatic variant profiles remains less clear. Owing to the introduction of PARP inhibitors and DNA damaging agents as therapies for germline *BRCA1/2*-mutant (*BRCA1/2*-MUT) advanced BC and germline/somatic *BRCA*-MUT ovarian cancer, the identification of non-familial BC cases with *BRCA*-like features is important, as these may represent a novel therapy opportunity (Tutt et al., 2018; Coleman et al., 2019; Vidula et al., 2020). Generally speaking, lacking the specific attention to the somatic mutations and effects in *BRCA1/2* genes on the molecular carcinogenic mechanism and progression in BC.

The objectives of this study were to discover genomic expression and immune patterns closely associated with the carcinogenesis of *BRCA* mutations, based on BC sequencing from The Cancer Genome Atlas (TCGA) database, and to identify the similarities and specificities underlying disease pathogenesis of somatic *BRCA*-mutant and wildtype BC. This was expected to provide novel insights into the molecular mechanisms influenced by this disease and *BRCA* mutations, and to identify putative targets for preventive strategies to treat individuals with this tumor susceptibility gene.

Triple-negative BC (TNBC) is a particular type of BC that is characterized by its heterogeneity, early recurrence, and poor survival (Foulkes et al., 2010). TNBC has an important relationship with *BRCA1/2*-MUT BC in terms of pathology and immunophenotype (Sharma et al., 2014; Belli et al., 2019). Therefore, given the lack of effective targeted therapies, the poor prognosis of TNBC patients, and the close relationship between *BRCA1/2*-MUT BC and TNBC, we also aimed to further identify closely related genes between these two subtypes, in order to reveal underlying molecular relations and promising therapeutic targets.

Some studies on BC have obtained important insights using high-throughput data and weighted gene co-expression network analysis (WGCNA) (Deng et al., 2019). WGCNA is used to construct scale-free gene co-expression networks and to find modules of genes with similar expression patterns under specific circumstances, which provides insights into key genes and signaling networks that could play critical roles in the progression of diseases by linking clinical phenotypes. However, as traditional WGCNA extensively used in many diseases studies, requires datasets of similar biological significance and analysis of only one group (one subtype), it may not be a suitable technique for analyzing differences in biological functions among several subtypes of diseases. Consensus WGCNA (Consensus network analysis) is rarely used in practice after it was proposed, but it has revealed changes in pathway and molecular mechanisms dependencies due to differential biological subtypes and achieved biologically meaningful results, in some studies such as the male-female mouse liver tissues, and human-canine osteosarcoma (Langfelder and Horvath, 2007; Jin et al., 2019; McDonough et al., 2019). Therefore, to identify the differences between *BRCA1/2*-MUT BC and *BRCA1/2* wild-type (*BRCA1/2*-WT) BC, we used consensus WGCNA, an improved analysis method which was conducted in our study using a slightly different R package code on the basis of the general principles of WGCNA, thereby perform the comparison of the effect of modules on associated clinical traits between mutant and WT cancers. Therefore, by Consensus network analysis, we have identified a series of important modules and genes within the modules, which have significantly universal/specific effects on the tumor stage, prognosis or metastasis indicators in *BRCA1/2*-MUT tumors, but some lacking obvious effects on wild-type tumors.

Immunophenotypes [estrogen receptor (ER); progesterone receptor (PR); human epidermal growth factor receptor 2 (HER2)] in BC, were of great significance for guiding clinical treatment and judging the treatment response, disease outcome, patient recurrence and prognosis. For instance, many studies demonstrated that Her-2 positive and TNBC subtypes had a poor prognosis and are more likely to relapse and metastasize early and frequently. The anatomic assessment of tumor size, regional lymph node involvement, and distant metastases (known as TNM), and metastasis indicators including number of positive lymph nodes, and cytokeratin, margin status as well, could provide the important predictors for the distinct clinical behavior and prognosis of BC (Hortobagyi et al., 2018). In our study, we focused on the above clinical traits of two BC subtypes, and performed the module-traits relationship analysis. This was followed by Gene Ontology (GO) and Kyoto Encyclopedia of Genes and Genomes (KEGG) enrichment, and ENCODE chromatin immunoprecipitation sequencing (ChIP-seq), to identify biological function and transcription factors (TFs) associated with modules. Highly connected genes within modules were identified and validated using TCGA and Gene Expression Omnibus (GEO) data, and survival analysis was performed, and shown to be *BRCA1/2-*associated critical regulatory genes. Furthermore, we identified *BRCA1/2*-MUT-specific immune patterns. Accordingly, our results would indicate common and specific patterns of regulation of tumor metastasis and the tumor immune microenvironment (TME) in BC, providing novel insights into the pathological process of this disease and the corresponding *BRCA* mutations.

## Materials and Methods

### Study Population

All data were downloaded from TCGA^1^. *BRCA1*-MUT and *BRCA2*-MUT BC samples were selected using cBioPortal^2^ (Gao et al., 2013) based on the presence of *BRCA1*/2 mutations and deep deletion copy number variation (CNV). We excluded BC samples with *BRCA1/2* amplification to avoid confounding factors. *BRCA1/2*-WT samples were randomly selected from BC patients without *BRCA* mutations or CNV; we also selected para-carcinoma tissues from our last study as normal samples (Li et al., 2020). Comparison of *BRCA1*-MUT and *BRCA2*-MUT samples with normal samples identified 4,889 and 5,124 differentially expressed genes (DEGs), respectively, using the EdgeR package. Differentially expressed RNA-sequencing expression profiles were used as an input for WGCNA, selected from more than 19,000 original profiles by keeping only the DEGs.

In the subsequent validation of the results, we used TNBC data from TCGA for internal validation and GEO datasets GSE3744 (Richardson et al., 2006; Alimonti et al., 2010) and GSE25307 (Jonsson et al., 2012) for external validation. The research subjects used in our work and relevant clinical traits information are shown in Table 1.

**TABLE 1:** Main subjects used in this study.

Analysis	Data source		Main subjects		
Exploration cohort	TCGA-BC		*BRCA1*-MUT (*N* = 36) vs. WT (*N* = 117)		
			*BRCA2*-MUT (*N* = 49) vs. WT (*N* = 117)		
		
			Its Clinical characteristics		
		
			*BRCA1*-MUT	*BRCA2*-MUT	WT
			Stage	Stage	Stage
			I (13.9%)	I (6.1%)	I (17.9%)
			II (75.0%)	II (65.3%)	II (55.6%)
			III (8.3%)	III (26.6%)	III (23.9%)
			IV (2.8%)	IV (2.0%)	IV (2.6%)
			Immunophenotype	Immunophenotype	Immunophenotype
			ER-(48.6%)	ER-(32.7%)	ER-(23.6%)
			PR-(56.8%)	PR-(49.0%)	PR-(32.1%)
			HER2-(64.8%)	HER2-(78.2%)	HER2-(84.5%)

Validation cohort	TCGA-TNBC		TNBC (*N* = 117) vs. normal (*N* = 112)		
	
	GEO dataset	GSE3744	*BRCA1*-associated cancer (*N* = 2) vs. normal (*N* = 7)		
		GSE25307	*BRCA1*-associated cancer (*N* = 34) vs. normal (*N* = 11)		
			*BRCA2*-associated cancer (*N* = 39) vs. normal (*N* = 11)		

### Weighted Gene Co-expression Network Analysis

Consensus WGCNA was conducted using the R package based on R version 3.5.1, to determine co-expressed genes between the two subtype groups from among the DEGs found in *BRCA1/2*-MUT BC. This was intended to identify key regulatory factors in the progression of *BRCA1/2*-MUT BC. We used fragments per kilobase of transcript per million fragments mapped (FPKM), or transcripts per million (TPM) normalization methods to standardize sequencing depth and gene length, in order to measure gene expression levels (Zhao et al., 2020). The MUT and WT expression profile data processed by FPKM were used for network construction. Hierarchical clustering of samples was used to identify outliers in each group (Figure 2). Based on the criterion of approximate scale-free topology, we chose the appropriate soft thresholding power for the function pickSoftThreshold; this is the power to which the co-expression similarity is raised to calculate adjacency (Supplementary Figure 3). We chose soft thresholding power values of 14 and 5, minimum module sizes 30 and 45, module detection sensitivity deepSplit 2, and cut height for merging of modules of 0.25, respectively, for *BRCA1*-MUT and *BRCA2*-MUT BC. Co-expression genes were assigned to modules via dynamic minimum tree-cutting arithmetic. Consensus network analysis was performed between MUT and WT patients (Supplementary Figure 4; refer to the research of Horvath et al., could get more detailed method introduction) (Langfelder and Horvath, 2007).

Identifying consensus modules significantly correlated with clinical traits in the MUT and WT subtypes, and with the same direction of correlation (positive or negative), could help to determine important modules correlated with clinical traits, including ER/PR and tumor stage. Upregulation and downregulation of each consensus module was determined based on the log2 fold change (log2FC) value: the mean gene expression in the mutant group was divided by the corresponding gene expression in normal tissue, and the log (base 2) of this value was taken. Module membership was determined by Pearson correlation between gene expression profiles and modules and used to rank module connectivity, in order to identify genes with high module membership in modules of interest, as well as those with high significance for traits.

### Enrichment Analysis for Biological Functions and Pathways

Module biological function was determined using g:Profiler^3^ to analyze GO and signaling pathway enrichment. GO analysis was performed with respect to biological process, cellular component, and molecular function (Raudvere et al., 2019). Comprehensive biological pathway analysis was conducted using the online KEGG pathway, Reactomen, and WikiPathways resources. Enriched GO terms and pathways were considered to be significant according to the criterion of adjusted *P* < 0.05.

### Identification of Associated Transcription Factors

Transcription factors for clinically significant modules (turquoise, purple, red, blue, turquoise, black, and brown) were identified using the iRegulon V.1.3 plugin in Cytoscape, based on 1120 ChIP-seq data from the ENCODE database, using the criterion of normalized enrichment score (NES) > 4.0 (Janky et al., 2014; McDonough et al., 2019).

### Identification and Validation of Candidate Genes in the Cancer Genome Atlas and Gene Expression Omnibus Database

We identified highly connected genes within modules as candidate genes to analyze the changes in transcriptional expression levels in the *BRCA1*-MUT, *BRCA2*-MUT, and WT groups compared with normal samples. In the validation cohort, we performed DEG analysis between TNBC expression profiles and para-cancerous tissues to achieve internal validation of candidate genes; GSE3744 (Richardson et al., 2006; Alimonti et al., 2010) and GSE25307 (Jonsson et al., 2012) were downloaded for external validation, using the limma package and *P* < 0.05 was taken as the criterion for a significant difference. Furthermore, we analyzed and evaluated the mRNA expression levels of candidate genes using an R package combined with standardized TPM expression data for several subtype groups (sub-division into three groups with TNBC included or excluded) to reflect the potential effects of TNBC subtypes on the final results.

### Survival Analysis of Candidate Genes for Breast Cancer

Candidate genes were evaluated with respect to their prognostic value in BC. We used Kaplan–Meier Plotter^4^, an online resource for survival analysis. As there were insufficient *BRCA1/2*-MUT BC cases for accurate survival analysis, the overall survival (OS) of all BC patients (*N* = 1879, from TCGA and GEO datasets) was analyzed using the Kaplan–Meier method, based on the classification of patients into high and low groups according to the mRNA expression of corresponding candidate genes. In the survival analysis, significance was defined as log-rank *P* < 0.05.

### Correlation Analysis Between *BRCA1/2*-MUT BC and Infiltrating Immune Cells

The TIMER web server^5^ is a comprehensive resource for systematical analysis of immune infiltrates across diverse cancer types (Li et al., 2016, 2017). TIMER was used to perform correlation analysis between the expression levels of hub genes in our study and tumor purity, as well as levels of tumor-infiltrating immune cells including B cells, CD4 + T cells, CD8 + T cells, macrophages, neutrophils, and dendritic cells.

### Correlations of Infiltrating Immune Cell Proportions, Tumor Mutational Burden, and Neoantigen Load With Hub Gene Expression

Based on bulk RNA-seq matrix information, bioinformatics methods using deconvolution-based analysis were used to score immune cells and the immune microenvironment, and to estimate immune cell infiltration. CIBERSORT enables characterization of cell composition and score estimation for 22 immune cell types in various tissues, based on gene expression profiles (Newman et al., 2015). We used the open-source CIBERSORT resource to compare infiltrating immune cell proportions in BC types with and without *BRCA1/2* mutations (*BRCA1*-MUT, *BRCA2*-MUT, and *BRCA1/2*-WT), as well as in TNBC and normal tissues. Spearman correlation analysis was used to test the correlations between hub gene expression and immune infiltration in *BRCA1/2*-MUT BC. Relationships of gene expression with tumor mutational burden (TMB) and neoantigen load in all BC types were analyzed using the maftools R package with TCGA mutation data and the SangerBox database^6^, an online platform for TCGA patient data based on R (Hu et al., 2021; Zhang et al., 2021).

### Statistical Analysis

Statistical analyses were performed with GraphPad Prism 7 and SPSS version 18.0. Differences among three or more groups were determined by analysis of variance or Kruskal–Wallis test, and further differences between groups were analyzed by the Dunnett method or student–Newman–Keuls test. Survival analysis was performed using the Kaplan–Meier method with log-rank test. Correlations between gene markers of tumor-infiltrating immune cells and hub genes were obtained as Spearman’s rho values. *P* < 0.05 was considered to indicate statistical significance.

## Results

### Weighted Gene Co-expression Network Analysis and Key Module Identification

**FIGURE 1:**
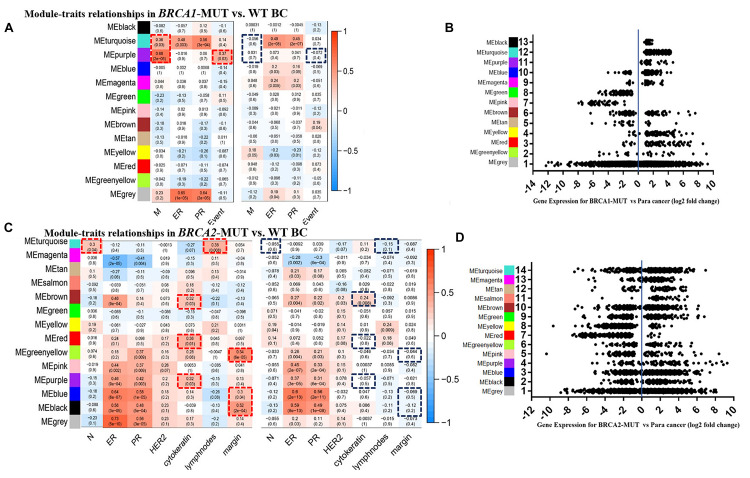
Modules associated with clinical traits in *BRCA1/2*-MUT and *BRCA1/2*-WT BC, and expression levels of corresponding module genes. **(A,C)** Identification of consensus modules for *BRCA1/2*-MUT and *BRCA1/2*-WT BC; complete figures are shown in Supplementary Figure 4. **(B,D)** Plot of gene expression levels for each consensus module of *BRCA1*-MUT and *BRCA2*-MUT BC to determine overall upregulation or downregulation of the module relative to normal tissue. N or M, the anatomic assessment of regional lymph node involvement or distant metastases. ER, status of estrogen receptor; PR, status of progesterone receptor; HER2, status of human epidermal growth factor receptor-2; lymph nodes, number of positive lymph nodes; ME, module eigengene. Negative ER, PR, or HER2 status was defined as “1” and positive status as “0.” Red frames, with significantly positive correlation in MUT; blue frames, lacking significant correlations in WT.

Consensus network analysis identified 12 consensus modules (genes not assigned to any of the modules are colored gray), including eight upregulated modules and three downregulated modules, in the *BRCA1*-MUT cohort compared with the WT cohort (Figures 1A,B); and 12 consensus modules including nine upregulated modules and two downregulated modules in *BRCA2*-MUT BC samples (Figures 1C,D; a complete module-trait heatmap is provided in Supplementary Figure 4).

**FIGURE 2:**
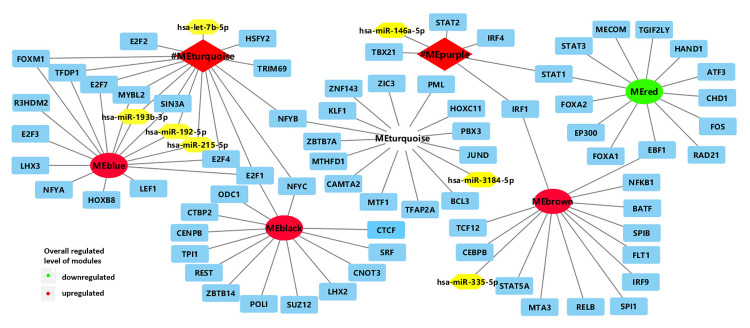
Network of enriched TFs (blue) and regulatory non-coding RNAs (yellow) with clinically significant modules (turquoise and purple modules in *BRCA1*-MUT BC; red, blue, turquoise, black, and brown modules in *BRCA2*-MUT). #ME represents modules from *BRCA1*-MUT samples. Red represents upregulated modules; green represents downregulated modules.

In the *BRCA1*-MUT group, ER/PR status was positively correlated with the turquoise module. Also, the turquoise and purple modules had significant correlations with tumor M stage, and the purple module had a significantly positive correlation with death, whereas no such correlations were found for WT samples (Figure 1A). But no obviously positive correlations were found for metastasis indicators in *BRCA1*-MUT samples (Supplementary Figure 4). These results suggested that genes in the turquoise and purple modules could explain the tumor metastasis mechanisms of *BRCA1* mutations and thus influence clinical prognosis. In *BRCA2*-MUT samples, ER/PR status was correlated with eight modules (magenta, brown, greenyellow, pink, purple, blue, and black). N stage was positively correlated with the turquoise module. Metastasis indicators, including cytokeratin, positive lymph nodes, and margin, were significantly correlated with the turquoise, brown, red, greenyellow, purple, blue, and black modules, but there were no such significant correlations in the WT group (Figure 1C).

### Enrichment Analysis for Biological Functions and Pathways

The turquoise and purple modules in the *BRCA1*-MUT group and the red, purple, blue, turquoise, black, greenyellow, and brown modules in the *BRCA2*-MUT group due to their correlations with clinical traits, were identified as clinically significant modules associated with carcinogenesis, progression, and metastasis of *BRCA1/2-*MUT BC. GO and pathway enrichment analyses were carried out for these modules (Table 2; complete results are shown in Supplementary Table 1).

**TABLE 2:** Biological functions (from GO and pathway enrichment) in clinically significant modules.

Sample	Module	Genes in module	Expression in disease	Biological function	
				Representative biological processes	Representative pathways
*BRCA1*-MUT	Turquoise	191	Up	Cell cycle; DNA replication	p53 signaling pathway; DNA double-strand break repair
	Purple	32	Up	Defense response to virus; response to type I interferon	JAK–STAT signaling pathway; cytokine-mediated signaling
*BRCA2*-MUT	Red	220	Down	Small molecule metabolic process; gluconeogenesis; cell migration	PPAR signaling pathway; AMPK signaling pathway; regulation of lipolysis in adipocytes
	Blue	1059	Up	Cell cycle; mitotic cell cycle; DNA repair	p53 signaling pathway
	Turquoise	1069		Organellar ribosome; mitochondrial; ribosome	COPI-dependent Golgi-to- endoplasmic reticulum retrograde traffic; mitochondrial translation
	Black	174	Up	Neurogenesis; anatomical structure morphogenesis	
	Brown	590	Up	Immune response; lymphocyte activation; regulation of cell–cell adhesion	Cytokine response; Cell adhesion molecules; JAK–STAT signaling; NF-κB signaling pathway; PD-L1 expression and PD-1 checkpoint pathway

In the *BRCA1*-MUT group, the turquoise module was enriched in cell cycle regulation, DNA replication, DNA damage response, and immune response; and the purple module was mainly enriched in processes related to the immune response, including defense response to virus and type I interferon signaling pathway, according to the GO enrichment analysis. Similarly, in the *BRCA2*-MUT group, modules were associated with the following categories: cell cycle, cell division, DNA replication, and DNA repair (blue module); cellular metabolic and catabolic processes (red module); cell adhesion and migration (red and brown modules); immune response (brown module); organ development (black module); and mitochondrial function (turquoise module). The purple and greenyellow modules showed no significant functional enrichment.

### Identification of Module-Associated Regulators

In the comparison of *BRCA1*-MUT and *BRCA1-*WT BC samples, 17 TFs and five microRNAs (miRNAs) were found to be enriched in the turquoise and purple modules (Figure 2 and Supplementary Table 2). The strongest enrichment scores were obtained for TFs associated with the upregulated purple immune response (STAT2, STAT1, IRF4, and IRF1) and the upregulated turquoise cell cycle module (E2F7, E2F4, E2F1, FOXM1, and TFDP1), with all NES > 9. Based on their expression levels in *BRCA1*-MUT BC relative to normal samples, many TFs were also significantly upregulated in the *BRCA1*-MUT group, including in the purple module [STAT1 (log2FC = 1.62) and IRF1 (log2FC = 1.10)] and turquoise module [E2F7 (log2FC = 3.16), E2F1 (log2FC = 2.83), FOXM1 (log2FC = 4.13), E2F2 (log2FC = 3.03), and MYBL2 (log2FC = 4.20)], suggesting a positive feedback loop in the regulation of *BRCA1*-associated carcinogenesis.

In the *BRCA2*-MUT samples, 77 TFs and five miRNAs were enriched in the six clinically significant modules (Figure 2 and Supplementary Table 2). The important TFs with high NES values were as follows: E2F4 in the (blue) cell cycle/DNA repair module; SPIB and IRF9 in the (brown) immune response module; and STAT1 and EZR, among others, in the modules associated with cell migration, adhesion, or angiogenesis (red and brown). Also, many TFs were also significantly upregulated in the *BRCA2*-MUT group, including E2F1 (blue module, log2FC = 3.13), FOXM1 (blue module, log2FC = 3.82), STAT1 (red module, log2FC = 1.82), ZIC3 (turquoise module, log2FC = 2.59), and IRF9 (brown module, log2FC = 1.30), again suggesting the existence of a positive feedback loop.

In summary, the turquoise module in the *BRCA1*-MUT group and the blue module in the *BRCA2*-MUT group both showed specific associations with regulation of the cell cycle and DNA repair, and had correlations with miR-193b, miR-192, and miR-215 and enrichment of the E2F family. The purple module in the *BRCA1*-MUT group and the brown module in the *BRCA2*-MUT group were associated with regulation of important immune response functions and inflammatory pathways, in particular, members of the IRF family including IRF1, IRF4, and IRF9, which are associated with immune regulation.

### Analysis of Clinically Significant Modules Including Functional Enrichment and Identification of Candidate Genes

**FIGURE 3:**
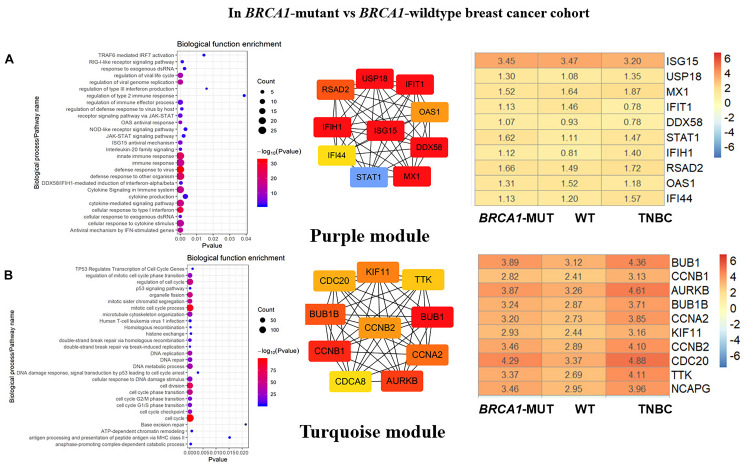
Biological function enrichment results for genes in clinically significant modules in *BRCA1*-MUT BC and gene network analysis. Biological function enrichment and corresponding gene information are shown for *BRCA1*-associated clinically significant modules. **(A)** Purple module, **(B)** Turquoise module. Left column: Top GO terms and KEGG pathways for genes from clinically significant modules. Middle column: Protein–protein interaction networks of top 10 most connected genes with highest degree in modules, constructed using STRING and Cytoscape. Right column: Heatmaps showing log2FC values for top 10 most connected genes. The ordinate is the gene name, and the abscissa (*BRCA1-*MUT, WT, and TNBC) represents different BC datasets from TCGA. Red represents log2FC > 0, blue represents log2FC < 0. Values are log2FC values of each gene in three sets, where FC is calculated as the mean gene expression within a group divided by the corresponding gene expression of normal tissues.

**FIGURE 4:**
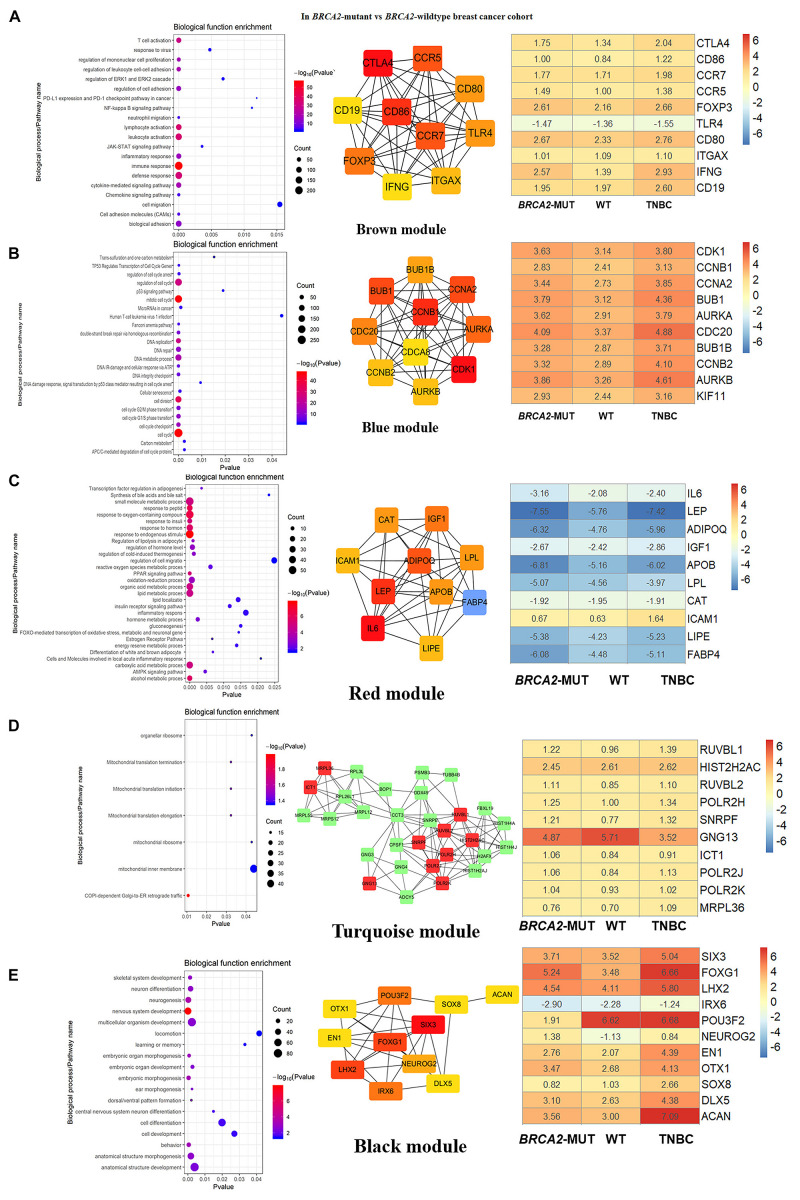
Biological function enrichment results for genes in clinically significant modules for *BRCA2*-MUT BC and gene network analysis. Biological function enrichment and corresponding gene information are shown for *BRCA2*-associated clinically significant modules. **(A)** Brown module, **(B)** blue module, **(C)** red module, **(D)** turquoise module, **(E)** black module. Left column: Top GO terms and KEGG pathways for genes from clinically significant modules. Middle column: Protein–protein interaction networks of top 10 most connected genes with highest degrees in modules, constructed using STRING and Cytoscape. Right column: Heatmaps showing log2FC values for top 10 most connected genes, similar to those shown in Figure 3.

The top 10 highly connected genes in each clinically significant module were screened for further analysis. As shown in Figures 3, 4, we determined the expression levels of these genes in the MUT, WT, and TNBC groups, as well in normal tissues for comparison. In several of the modules associated with immune response and cell cycle regulation, gene expression levels showed sequential changes from TNBC to mutant BC to WT BC. We found that some modules specifically associated with the clinical characteristics of *BRCA2*-MUT BC were related to tumor metabolism, cell migration, and other biological functions, and most candidate genes were also expressed in TNBC (Figure 4).

#### Immune Response

In both the *BRCA1*-MUT and *BRCA2*-MUT cohorts, we found upregulated modules significantly related to immune response (the purple and brown modules, respectively), indicating that genes in these modules may have specific effects on progression and metastasis of *BRCA1/2*-MUT BC but no significant impact on *BRCA1/2*-WT BC. In the *BRCA1*-MUT group, the purple module was enriched in processes involved in immune response, including defense response to virus and regulation of cytokine production. On the other side, the pathway enrichment analysis results suggested that genes in this module were mainly related to the RIG-I-like receptor signaling pathway, JAK–STAT signaling pathway, and NOD-like receptor signaling pathway (Figure 3A). The top 10 genes ranked by degree were identified as candidate genes. These candidate genes included *ISG15* and *STAT1* (Figure 3A). The top seven genes had the highest node degrees among the candidate genes (i.e., node degree 31). In the *BRCA2*-MUT group, the brown module was enriched in similar GO biological process and pathways (Figure 4A), and *CTLA4* had the highest node degree of 100.

#### Cell Cycle/DNA Repair

In the *BRCA1*-MUT group, the turquoise module was enriched in processes including cell cycle, cell cycle phase transition, and DNA repair (Figure 3B). Among the top 10 genes, which included *BUB1* and *CCNB1*, *BUB1* had the highest node degree (i.e., 150). In the *BRCA2*-MUT group, the blue module was enriched in similar GO biological processes and pathways (Figure 4B). Among the top 10 hub genes, which included *CDK1*, *CCNB1*, *CCNA2*, and *BUB1*, *CDK1* had the highest node degree of 141.

#### Cell Migration/Adhesion, Metabolic Processes, and Organ Development

Notably, the red and brown modules (Figures 4C,D) were found to specifically influence the progression and metastasis of *BRCA2*-MUT BC, via their important roles in the regulation of cell migration and adhesion. No such correlations were observed in WT BC. The red module was also enriched in metabolic processes including regulation of lipolysis, response to insulin, gluconeogenesis, inflammatory response, and regulation of cell migration. On the other side, pathway enrichment analysis suggested that the genes in the red module were mainly related to the PPAR signaling, AMPK signaling, and estrogen signaling pathways (Figure 4C). The turquoise module was mainly associated with mitochondrial translation functions (Figure 4D), and the genes in the black module appeared to have roles in anatomical structure morphogenesis and organ development (Figure 4E).

### Validation and Identification of Hub Genes

By internal validation using TCGA data, we demonstrated that most candidate genes were also significantly expressed in TNBC (Figures 3, 4). This indicated the accuracy of our results; the important roles of these genes in the progression of BC, especially in *BRCA1/2*-MUT BC; and the relationships between TNBC and *BRCA1/2* mutations at the molecular level.

**FIGURE 5:**
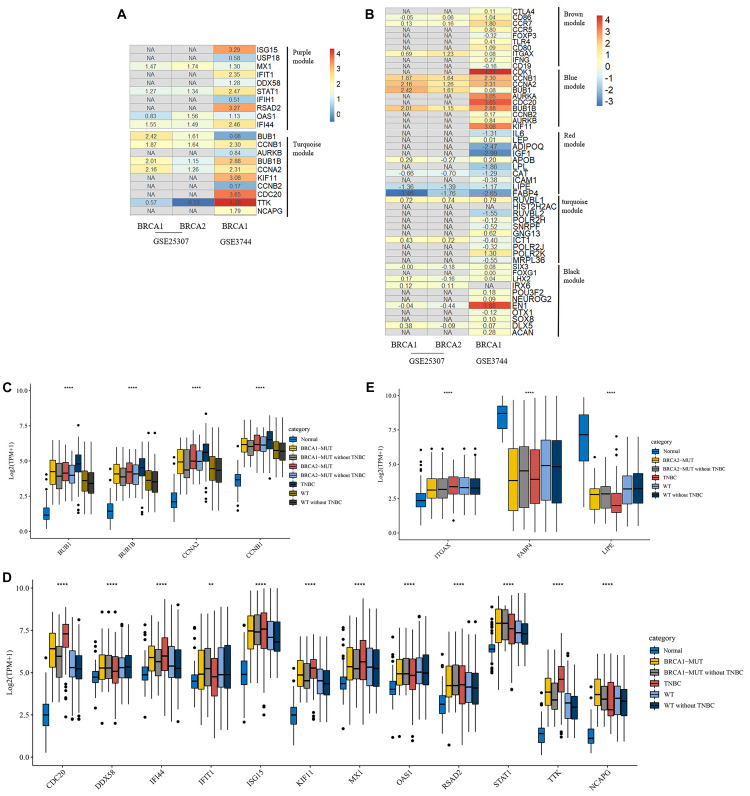
Heatmap of log2FC values for external validation of hub genes using GEO datasets and transcriptional expression levels of validated hub genes. **(A,B)** Heatmaps of log2FC values for hub genes in *BRCA1*-MUT **(A)** and *BRCA2*-MUT **(B)** BC cohorts for GEO external validation. The ordinate is the gene name and the abscissa represents different groups in GEO datasets. Values are log2FC values of each gene in three sets, where FC is calculated as the mean gene expression in the relevant group divided by the corresponding gene expression in normal tissue. “NA” represents GEO datasets that did not contain these genes. **(C–E)** Expression levels of validated hub genes in *BRCA1*-MUT **(C,D)** and *BRCA2*-MUT **(C,E)** BC. To show the potential effects of TNBC subtypes on BC, the hub gene expression was analyzed in multiple groups: normal (*N* = 112), *BRCA1*-MUT BC (*N* = 36), *BRCA1*-MUT BC excluding TNBC (*N* = 26), *BRCA2*-MUT BC (*N* = 49), *BRCA2*-MUT BC excluding TNBC (*N* = 42), TNBC (*N* = 116), WT (*N* = 117), and WT excluding TNBC (*N* = 103). **P < 0.01, ****P < 0.0001.

Moreover, we used GEO datasets GSE3744 and GSE25307 for further validation of these hub genes. As shown in Figure 5, there were many genes that were not found in the GEO datasets, and most other genes had differential expression relative to normal tissues. In *BRCA1*-MUT BC, genes including *BUB1*, *CCNB1*, *BUB1B*, *CCNA2*, and *TTK* showed consistently upregulated expression in at least one of the validation datasets. In *BRCA2*-MUT BC, *ITGAX*, *CCNB1*, *CCNA2*, *BUB1*, and *BUB1B* also showed upregulated expression, whereas *LIPE* and *FABP4* were downregulated.

Given the different immune profile of the TNBC subtype compared with other BC subtypes, and the associations between *BRCA1/2* mutations and TNBC, we selected non-TNBC tissues with *BRCA1/2* mutations and non-TNBC *BRCA1/2*-WT tissues (that is, MUT and WT samples excluding all TNBC subtypes) for comparison. As shown in Figures 5C,D, *BRCA1*-associated hub genes *BUB1*, *BUB1B*, *CCNB1*, *CDC20*, *IFI44*, *ISG15*, *KIF11*, *STAT1*, *RSAD2*, *TTK*, and *NCAPG* showed upregulated gene expression. Similarly, *BRCA2*-associated hub genes *BUB1*, *BUB1B*, *CCNB1*, *FABP4*, and *LIPE* showed differential mRNA expression levels in *BRCA2*-MUT and non-TNBC *BRCA2*-MUT tissues, suggesting that TNBC-subtype effects did not completely prevent the identification of many hub genes. It is worth noting that in the TNBC subtype, some oncogenes, including *BUB1*, were further upregulated at the transcriptional level, whereas tumor suppressor genes *FABP4* and *LIPE* were downregulated, compared with normal tissues, *BRCA1/2*-WT, and *BRCA1/2* mutants.

**FIGURE 6:**
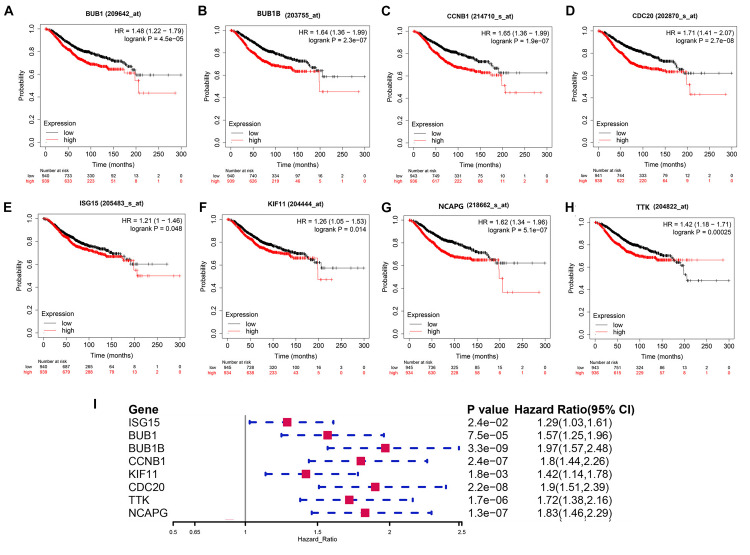
Prognostic significance of validated hub genes in BC. **(A–H)** Validated hub genes with significant prognostic value for OS in BC are shown: **(A)** *BUB1*, **(B)** *BUB1B*, **(C)** *CCNB1*, **(D)** *CDC20*, **(E)** *ISG15*, **(F)** *KIF11*, **(G)** *NCAPG*, and **(H)** *TTK*. **(I)** Prognostic value of these eight hub genes for 5-year OS in BC.

In this study, we found that *BUB1*, *BUB1B*, *CCNB1*, *CDC20*, *ISG15*, *KIF11*, *NCAPG*, and *TTK* were not only significantly expressed in corresponding tumor tissues with *BRCA* somatic mutations compared with WT BC and normal tissue but also showed significant prognostic value for BC (Figures 6A–I). Therefore, these validated genes are potential key hub genes and critical regulatory factors in *BRCA1*/*2*-MUT BC, which could represent new prognostic or diagnostic biomarkers and provide novel insights into the mechanisms of *BRCA1/2* mutations in cancer development.

### Correlation Analysis Between Hub Genes of *BRCA1/2*-MUT BC and Infiltrating Immune Cells, Tumor Mutational Burden, and Neoantigen Load

We found that the validated hub genes could influence metastasis of *BRCA1/2*-MUT BC through mechanisms including regulation of immune response, cell cycle, cell migration, and metabolic processes. Many recent studies have demonstrated that tumor-infiltrating immunocytes (TICs) can regulate and participate in the TME, regulate tumor progression, and influence lymph node metastasis and prognosis (Azimi et al., 2012; Yusuf et al., 2020). Thus, we used the TIMER web server and CIBERSORT to assess immune infiltration and its correlations with *BRCA1*/*2-*associated hub genes.

**FIGURE 7:**
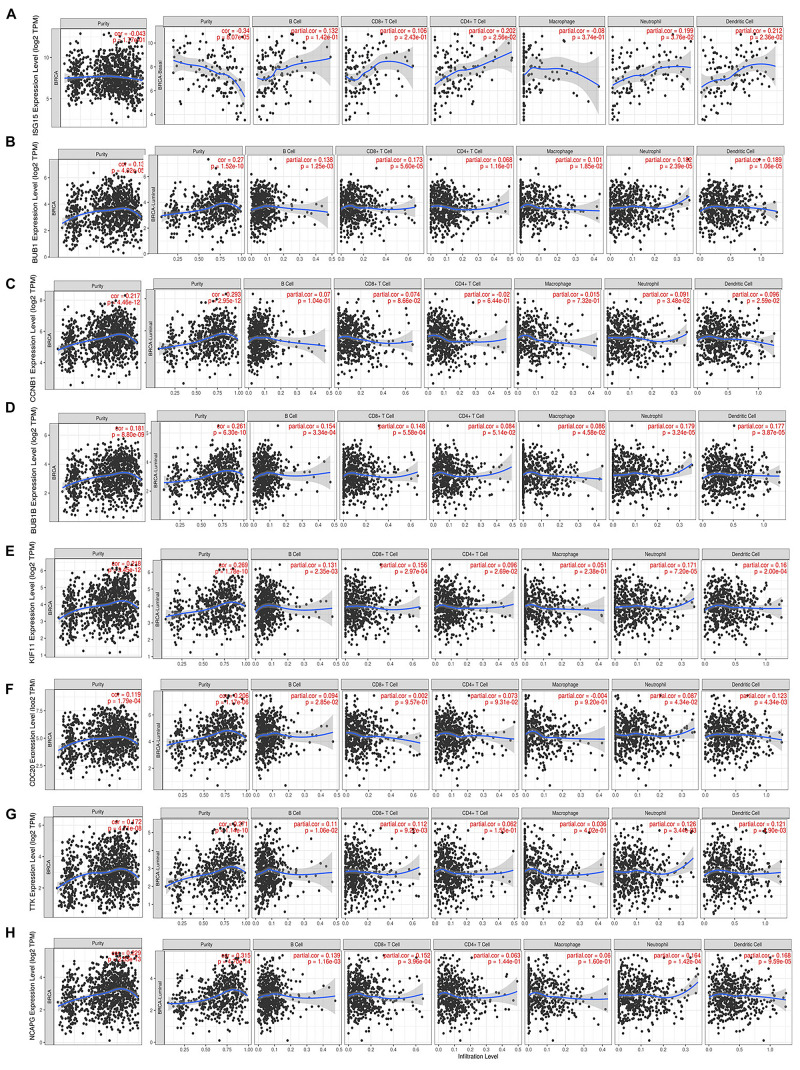
Correlation of each hub gene’s expression with immune infiltration levels in BC. Complete correlation analysis results for BC subtypes are given in Supplementary Figure 5. Correlation of *ISG15* **(A)**, *BUB1* **(B)**, *CCNB1* **(C)**, *BUB1B* **(D)**, *KIF11* **(E)**, *CDC20* **(F)**, *TTK* **(G)**, and *NCAPG* **(H)** expression with TICs.

We noted that hub gene expression was positively correlated with infiltrating immune cells, including T cells, neutrophils, and dendritic cells, in almost all BC subtypes (basal-like, luminal, and HER2-positive; Figure 7 and Supplementary Figure 5), of which the basal-like and luminal subtypes showed significant associations with tumor purity. *ISG15* expression was negatively correlated with tumor purity in basal-like BC, as well as with levels of infiltrating T cells, neutrophils, and dendritic cells; its correlation with tumor purity in the luminal subtype was undefined (Figure 7A). However, *BUB1*, *CCNB1*, *BUB1B*, *KIF11*, *CDC20*, *TTK*, and *NCAPG* (Figures 7B–H), which are closely related to regulation of the cell cycle and DNA repair, showed strong positive correlations with tumor purity in luminal-type BC.

**FIGURE 8:**
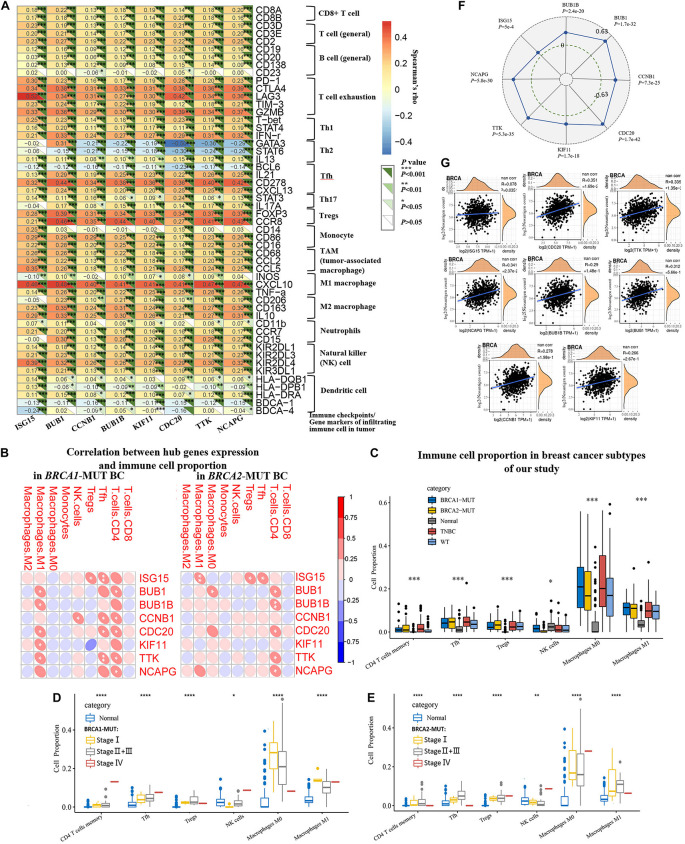
Analysis and identification of *BRCA1/2*-MUT-specific immune-associated patterns and their relationships with TMB and neoantigen load in BC. **(A)** Correlation analysis between related hub genes (*ISG15*, *BUB1*, *CCNB1*, *BUB1B*, *KIF11*, *CDC20*, *TTK*, and *NCAPG*) and immune cell markers in TIMER. Expression scatter plots for pairs of genes adjusted by tumor purity in BC (1093 cases), together with Spearman’s rho value and estimated statistical significance, reflecting the correlations between hub genes and immune checkpoints/gene markers of immune cells. **(B)** Correlations between hub gene expression and infiltrating immune cell proportions in *BRCA1*-MUT and *BRCA2*-MUT BC. **(C)** Infiltrating immune cell proportions (with obvious correlations with hub gene expression in **(B)** in *BRCA1/2*-MUT BC, TNBC, *BRCA1/2*-WT BC, and para-cancerous normal tissues, using CIBERSORT package. **(D,E)** Comparison of immune cell proportion between stages I, and stage II + III and stage IV in *BRCA1/2*-MUT BC patients, and para-cancerous normal tissues. **(F)** Correlations between hub gene expression and TMB in BC. **(G)** Correlations between hub gene expression and neoantigen load in BC. *P < 0.05, **P < 0.01, ***P < 0.001, ****P < 0.0001.

Furthermore, to explore the effects of hub genes on TICs, we analyzed the relationships between their expression levels and various specific gene markers of immune cells, including innate immune cells and adaptive immune cells, after adjustment for tumor purity (Figure 8A and Supplementary Table 3). As shown in Figure 8A, we first determined the correlation coefficients between the expression levels of hub genes (*ISG15*, *BUB1*, *CCNB1*, *BUB1B*, *KIF11*, *CDC20*, *TTK*, and *NCAPG*) and TIC markers. Positive correlations with hub gene expression were observed for markers of Th1 cells including T-bet, STAT4, and IFN-r; markers of T regulatory cells (Tregs) including FOXP3 and CCR8; and markers of Tfh cells including IL21, CD278, and CXCL13. Markers of natural killer cells (NK cells), tumor-associated macrophages, and M1/M2 macrophages also showed possible positive correlations with expression of hub genes. We observed significant positive correlations with markers of T cell exhaustion, including FOXP3, PD-1, CTLA4, LAG3, TIM-3, and GZMB. We also found that *BRCA1/2*-specific infiltration patterns displayed a consistent lack of correlation with Th2 and Th17 cell markers.

Comparison of cell proportions between *BRCA1/2*-MUT BC and normal tissues, and their correlations with hub genes (Figures 8B,C), also partially supported the idea that *BRCA1/2* mutations could increase proportions of infiltrating immune CD4+ T cells, for instance, Th1, Tfh, and Treg cells, in BC. Specifically, the expression levels of some hub genes were positively correlated with levels of infiltrating CD4+ T cells, Tfh cells, Tregs, and M1 macrophages (Figure 8B). We also observed significantly higher levels of immune infiltration in *BRCA1/2*-MUT BC, especially for CD4+ T cells, Tfh cells, Tregs, and M1 macrophages, compared with normal tissue or *BRCA1/2*-WT BC (Figure 8C). This was followed by the further analysis of the above infiltrating immune cells proportions in different clinical stage of tumors. The results show that Tfh cells and Tregs have a higher proportion in patients with high tumor stage than in stage I, but decreased level trend of M0, M1 macrophages infiltration, compared with that of stage I tumors (Figures 8D,E), suggesting that with the progression of *BRCA1/2*-mutant BC, the proportions of infiltrating immune cells showed an increased trend, especially Tfh and Tregs.

The high TMB and neoantigen load in tumors favor the infiltration of immune effector cells. Antitumor immunotherapy responses are strong in these patients relative to others (Picard et al., 2020). In many cancer types, higher TMB may be associated with favorable response to anti-PD-1/PD-L1 immunotherapy (Wang and Li, 2019). In our study, higher expression levels of all hub genes were positively associated with TMB in BC patients (Figure 8F). *ISG15*, *CDC20*, *TTK*, and *NCAPG* expression showed positive correlations with neoantigen load in TCGA BC patients (Figure 8G). These results suggest that the hub genes, especially *ISG15*, *CDC20*, *TTK*, and *NCAPG*, could predict the efficacy of immunotherapy in BC.

Therefore, these complementary results demonstrate strong positive correlations of *BRCA1/2*-associated hub genes with T cell infiltration and T cell exhaustion, especially that involving T cell subtype markers such as Th1 and Tfh cells and Tregs. These findings suggest that *BRCA1/2* mutation of breast tumors could recruit immune cells in the TME, resulting in the recruitment of Th1 cells, Tfh cells, and Tregs, and T cell exhaustion.

## Discussion

Breast cancer remains the most common cancer affecting female patients and a major cause of cancer-related deaths among women worldwide (DeSantis et al., 2014). There are ongoing efforts to assess the mutational profiles of BC. Somatic mutations in *TP53* and *PIK3CA* occur at rates of about 25% (Azim and Partridge, 2014; Weisman et al., 2016); on the other hand, there are numerous novel subtype-associated genetic mutations, including specific mutations of *GATA3*, *PIK3CA*, and *MAP3K1* in the luminal A subtype (Cancer Genome Atlas Network, 2012). However, genetic evidence regarding mutation profiles and their implications for female patients remain to be clarified. There were few related studies about explaining the influence of specific tumor somatic mutation types on the molecular portraits in BC. Owing to the need to explore prognostic or predictive markers and therapeutic targets for diseases, the prevailing research strategies include use of large-scale genome sequencing data combined with bioinformatic analyses (Wu et al., 2019). The transcriptome landscapes of specific subtypes of tumors represent a promising area of study; given the trend of tumor precision medicine, this has become an important direction of bioinformatics research.

In this study, we constructed a consensus co-expression gene network of DEGs influenced by *BRCA1*/*2* mutations and associated TFs, based on results from our previous study (Li et al., 2020). The aim of the present study was to explore the regulatory factors and immune-associated patterns that have important common and specific influences and are involved in critical regulation of *BRCA1/2* mutations. This was expected to improve our understanding of the molecular mechanisms of BC and provide insights for use in individualized treatment. Consensus WGCNA has rarely been used in research on this related subject. Consensus WGCNA enabled us to focus on the differences between the several subtypes with respect to transcriptome characteristics associated with clinical traits, in order to identify the similarities and differences between several subtypes of BC associated with tumor somatic mutations or prognosis/metastasis types.

The two disease subtypes (genetic mutation and wild type) were biologically very similar, but significant differences were observed, including multi-gene and multi-signal differences, as demonstrated by consensus network analysis in our study. Consensus network analysis identified the specific and common carcinogenic mechanisms involving *BRCA1/2* mutations in BC with relation to differential clinical traits. For example, we found that two modules associated with immune response and cell cycle regulation were significantly correlated with clinical traits (tumor M stage and poor prognosis) in *BRCA1*-MUT BC, but without significant influence in WT BC, suggesting that the modules and associated genes influenced by *BRCA1* mutations could regulate and cause dysfunction of the cell cycle and immune response, and thus influence the metastasis and prognosis of *BRCA1*-MUT BC. In *BRCA2*-MUT BC, the critical co-expression modules were significantly positively correlated with N stage, cytokeratin, number of positive lymph nodes, and margin indicators but lacked significant correlations in WT BC. Therefore, we identified these modules as *BRCA2*-MUT-type-specific co-expressed modules. As number of positive lymph nodes, cytokeratin, and margin indicators are important clinical prognostic factors, as demonstrated by many studies, these results suggested that *BRCA*1/2 could influence the genes in these modules and thus affect clinical outcomes (Engel et al., 2019). In addition, specific *BRCA2-*associated modules showed obvious effects on regulation of the cell cycle, DNA repair, cell adhesion/migration, immune response, and cellular metabolic processes via enriched pathways including p53 signaling, PPAR signaling, the JAK–STAT pathway, and the PD-1 checkpoint pathway.

Our results were consistent with some of the key functions of BRCA proteins, including cell cycle transition and the DNA damage response, as demonstrated by previous studies. For instance, *BRCA1* mutations have been shown to trigger deregulation of genes involved in the G2/M cell cycle transition, whereas *BRCA2* mutations could cause deregulation of genes involved in the G1/S cell cycle checkpoint (Bouwman and Jonkers, 2012; Vaclova et al., 2015; Tanaka et al., 2018). However, there was a lack of research evidence for other enriched biological processes and signaling pathways identified in our study, such as immune response and the JAK–STAT pathway.

Cytokeratin and tumor margin indicators are essential for the development and metastasis of various cancers and are regarded as important predictors of distant metastasis risk and poor prognosis (Lee et al., 2019; Li et al., 2019). A recent study found that *BRCA1/2* carriers frequently experienced lung, distant lymph node, and central nervous system involvement (Song et al., 2020). In our study, the co-expressed gene network displayed stronger relationships with positive lymph nodes, cytokeratin, and margin indicators, compared with WT BC, suggesting that hub genes influenced by BRCA proteins could more effectively promote metastasis. Regarding the possible molecular mechanisms underlying this effect, our results suggest that *BRCA1* mutations could influence cell cycle transition and tumor immunomodulation through the p53 or JAK–STAT pathway, whereas *BRCA2* mutations could influence cell cycle transition, tumor immunomodulation cell migration, and energy metabolic processes via PPAR signaling, AMPK signaling, p53 signaling, the JAK–STAT pathway, the NF-κB pathway, and the regulation of cell adhesion molecule interactions. These effects could be related to metastasis risk and poor prognosis in BC patients.

Furthermore, regulators cross-linking these modules include the transcription factors STAT1, IRF1, E2F7, E2F1, E2F2, FOXM1, and MYBL2 which were significantly upregulated in *BRCA1*-MUT BC, and E2F1, FOXM1, STAT1, ZIC3, and IRF9 upregulated in *BRCA2*-MUT BC. These results suggested the existence of a positive feedback loop involving regulatory factors but also indicate potential therapeutic targets.

The ultimate goal of precision medicine is to identify specific molecular alterations that permit application of effective targeted drugs (Belli et al., 2019). Many studies have focused on the relationships between *BRCA* mutations and TNBC, and *BRCA1/2* mutations are expected to provide potential therapeutic targets for TNBC. In this respect, although PARP inhibitors have been approved for the treatment of *BRCA*-MUT and invasive BC in HER2-negative patients, the emergence and development of drug resistance is inevitable. Therefore, more research on therapeutic targets is needed with respect to the precision treatment of TNBC and *BRCA*-MUT BC. The genes influenced by *BRCA* mutations will provide novel insights into the pathological processes underlying disease mechanisms in *BRCA*-MUT BC and guide therapeutic strategies. Notably, the highly connected genes identified in our study, denoted hub genes, showed sequential changes in expression levels from TNBC to *BRCA1/2*-MUT BC to *BRCA1/2*-WT BC, suggesting close relationships between *BRCA1/2* mutations and TNBC at a molecular level. Furthermore, by validation using GEO datasets and survival analysis, we identified hub genes *BUB1*, *CCNB1*, *BUB1B*, *ISG15*, *KIF11*, *CDC20*, *TTK*, and *NCAPG*, which act as important regulatory genes in the progression of *BRCA1/2*-MUT BC.

In fact, some studies have indicated that *BUB1* (Takagi et al., 2013), *KIF11* (Pei et al., 2017), *TTK* (Sugimoto et al., 2017) and so on (Yuan et al., 2006) might be involved in tumorigenesis and become new candidate biomarkers for BC or TNBC treatment. Recent studies found that *ISG15* could become markers predicting the metastasis in TNBC (Rojas et al., 2019). *NCAPG* was a newly found markers associated with the prognosis of BC (Xiao et al., 2020), but its mechanism and more evidence needed to be further reported. And the important functions of these genes in *BRCA1/2*-mutant BC were still lack of relevant studies. In our study, the upregulated expression and prognostic value of these hub genes in *BRCA1/2*-mutant BC suggested that its roles in this type of breast cancer were equally noteworthy. More importantly, we found these hub genes were closely associated with higher levels of immune infiltration in *BRCA1/2*-mutant BC, especially CD4+ T cells, Tfh cells, Tregs, and M1 macrophages and T cell exhaustion. *ISG15*, *CDC20*, *TTK*, and *NCAPG* expression showed positive correlations with tumor mutational burden and neoantigen load in BC patients. Our study indicated the importance of further exploration of these hub genes with potential to recognize immune infiltrated and predict the efficacy of immunotherapy.

Characterizing the mechanistic relationships of cancer with inflammation and the immune microenvironment can be conducive to identifying novel individualized biomarkers or therapeutic targets. The TME is essential in the development and progression of cancer. Immune response and inflammation are classic and prevalent examples of functions of the TME. Moreover, evasion of immune destruction and tumor-promoting inflammation are essential hallmarks of cancer (Hanahan and Weinberg, 2011; Iyengar et al., 2016; Zhao et al., 2019). The relationships among immune response, inflammation, and BC are complex and important. The roles of TILs within the immune microenvironment have received increasing attention because of their important regulatory effects on the progression, metastasis, and prognosis of tumors. Our functional enrichment results demonstrated that multiple genes affected by *BRCA1/2* mutations showed significant enrichment in immune response and lymphocyte activation in BC. Some other studies reported that TNBC was the most immunogenic subtype and attracted TILs owing to its genomic instability and higher mutation rate (Oshi et al., 2020). However, the global characteristics of the immune response and inflammation in BC with *BRCA1/2* mutations remained unknown.

Our study indicated a meaningful positive correlation of *BRCA1/2*-associated hub genes with infiltration of T cells (Th1, Tfh, and Treg cells) and T cell exhaustion. Engel et al. (2014) reported that infiltration of Tregs was significantly higher in TNBC or in BC with mutation of *BRCA1*. A recent study indicated that Treg frequencies increased with nodal invasion, and that Treg-mediated local immunosuppression might influence tumor-draining lymph node invasion by metastatic cells and lead to poor prognosis (Núñez et al., 2020). There is also evidence that Tregs may negatively regulate responses of CD8+ T cells and natural killer cells to tumor cells, as well as leading to severe neovascularization and metastasis, together with the effects of Tfh and B cells, as a pro-tumor immune response (Marshall et al., 2016). Many studies have shown that Th1 cells play a critical part in anti-tumor immunity through inducing and directing effector cellular immune responses as well as inflammatory responses, together with their cytokines (especially IFNγ), in several solid tumor types (Faghih et al., 2014). It is discovered that the original Tfh cells in BC promote the accumulation of Tregs in the tumor, inhibit the anti-tumor immunity, and ultimately promote the development of BC (Gu-Trantien et al., 2017). Notably, our study also revealed a series of further and detailed immune infiltration information influenced by *BRCA1/2* mutation type in BC. Our findings implied that *BRCA1/2* mutations could regulate multiple genes and pathways as described above, resulting in specific immune patterns, with effects on the TME including activation and infiltration of Tregs, Th1 cells, and Tfh cells, as well as T cell exhaustion. Therefore, our study demonstrates a potential mechanism by which *BRCA1/2* could regulate T cell function and influence infiltrating immune cell recruitment and functional suppression in the TME. Furthermore, our findings indicated that during the development of *BRCA1/2*-mutant BC, Tfh cells and Tregs played a tumor-promoting role, and their proportions showed increased levels, suggesting the possibility that immune escape associated with the regulation of Tregs was aggravated, and the tumor’s ability to fight the immune response might further strengthened. However, we also observed the proportions of NK cells, M1 macrophages with the potential to exert an antitumor effect showed dynamic changes, which indicate the opposition between antitumor immune cells and tumor-promoting immune cells at a proportional level, and the escalating immune confrontation in the progression of *BRCA1/2*-mutant BC.

One important limitation of the study was that the *BRCA1/2*-MUT BC data did not distinguish between somatic mutations and germline mutations of *BRCA* genes. Therefore, we were unable to confirm whether the findings would be applicable to hereditary BC patients. Although duplicate verifications using TCGA and GEO data were performed to identify more credible key genes, further molecular biological experiments are required to support this investigation and confirm our results.

## Conclusion

Our data demonstrated enrichment of several pathways associated with *BRCA* mutations; these results were partly consistent with current knowledge of the functions of *BRCA*. We identified a correlation gene expression network and associated TFs in *BRCA1/2*-MUT BC to provide comprehensive and novel insights into the pathological mechanisms involved in *BRCA1/2*-MUT BC and the close relations between *BRCA1/2* mutations and TNBC at a molecular level. Our study identified several candidate regulatory factors and hub genes as promising targets for intervention, including members of the E2F TF family and IRF family. Moreover, *BUB1*, *CCNB1*, *BUB1B*, *ISG15*, *KIF11*, *CDC20*, *TTK*, and *NCAPG* showed prognostic value and potential roles in assessment of the efficacy of BC immunotherapy. These results indicate the close relationships between *BRCA1/2* mutations and TNBC at the molecular level. Furthermore, we identified immune-associated patterns in *BRCA1/2*-MUT BC, which manifested as the recruitment of T cells, including Th1 and Tfh cells, Treg infiltration, and T cell exhaustion; these patterns would explain the critical influence on tumor progression and metastasis of *BRCA1*/*2* mutations in BC via common and specific TME regulation patterns.

## Data Availability Statement

The datasets presented in this study can be found in online repositories. The names of the repository/repositories and accession number(s) can be found in the article/Supplementary Material.

## Author Contributions

YL and QH conceived and designed the experiments. WD collated and checked the data. YL and XZ performed the consensus WGCNA network. PZ and TZ performed the function enrichment analysis. LM, MQ, and PZ helped to perform the analysis about tumor-infiltrated immune cells. XM explained and collected the content about *BRCA1/2* mutations. YL was responsible for bioinformatics and bio-statistics analysis, and wrote the manuscript. QH revised the manuscript. All authors contributed to the article and approved the submitted version.

## Conflict of Interest

The authors declare that the research was conducted in the absence of any commercial or financial relationships that could be construed as a potential conflict of interest.

## Publisher’s Note

All claims expressed in this article are solely those of the authors and do not necessarily represent those of their affiliated organizations, or those of the publisher, the editors and the reviewers. Any product that may be evaluated in this article, or claim that may be made by its manufacturer, is not guaranteed or endorsed by the publisher.

## Footnotes

1
https://portal.gdc.cancer.gov/

2
http://www.cbioportal.org/index.do

3
https://biit.cs.ut.ee/gprofiler

4
http://kmplot.com/analysis/

5
https://cistrome.shinyapps.io/timer/

6
http://www.sangerbox.com/
