# Supplementary material for: Comprehensive Analysis of Regulatory Factors and Immune-Associated Patterns to Decipher Common and BRCA1/2 Mutation-Type-Specific Critical Regulation in Breast Cancer
Source: Front Cell Dev Biol. 2021 Oct 18;9:750897. doi: 10.3389/fcell.2021.750897 (PMC8558486; doi:10.3389/fcell.2021.750897)

Figure S1 Venn analysis were used to identify all common genes from differentially expressed genes of *BRCA1/2*-mutant breast cancer compared with paracancer tissues. Comparison of *BRCA1*-mutant breast cancer and para-carcinoma tissue identified 4889 differentially expressed genes, and 5124 in *BRCA2*-mutant BC patients. Therefore, venn analysis showed 6013 all common genes.

Figure S2 Hierarchical clustering of samples were used to identify outliers in each group, in order to perform further consensus weighted gene co-expression network analysis (WGCNA). Red lines on dendrograms refer to the threshold that was set for identifying outliers. We identified 1 outlier in wild-type group. MUT BC, *BRCA1/2*-mutant breast cancer; WT BC, wild-type breast cancer.

Figure S3 Summary network indices (y-axes) as functions of the soft thresholding power (x-axes). Numbers in the figure S3 indicated the corresponding soft thresholding powers. The plots indicated that approximate scale-free topology is attained around the soft-thresholding power of 14 or 5 for *BRCA1*-mutant or *BRCA2*-mutant set vs wild-type breast cancer. Because the summary connectivity measures decline steeply with increasing soft-thresholding power, it is advantageous to choose the lowest power that meets the approximate scale-free topology criterion.

Figure S4 Complete module–trait comparisons based on consensus modules identification, following the mentioned process of figure S2-S3.
Relationships of consensus module eigengenes and clinical traits in the MUT data and WT data. Each row in the table corresponds to a consensus module, and each column to a trait. Numbers in the table report the correlations of the corresponding module eigengenes and traits, with the p-values printed below the correlations in parentheses. Figure S4A-B, consensus modules identification for *BRCA1*-mutant and wild-type breast cancer. Same genes were put in the same module for both groups. E-F, consensus modules identification for *BRCA2*-mutant and wild-type breast cancer. Same genes were put in the same module for both groups. Figure S4C and S4G, modules significantly correlated to clinical traits in MUT and WT groups and in the same direction (positive or negative correlation), were chosen the correlation that has the lower absolute value and the opposite relationships were defined as zero relationship (NA), thus to form the module-trait relationships that summarizes the two groups into one measure. Figure S4D and S4H, plot of fold change of genes in each consensus module of *BRCA1/2*-mutant breast cancer to determine overall upregulation or downregulation of the module relative to corresponding para cancerous tissue.

Figure S5 Correlation of each hub gene expression with immune infiltration level in different subtypes of breast cancer.
Note and significance: For immune response-associated hub genes, ISG15, IFIT1, MX1, DDX58, STAT1, RSAD2, OAS1, IFI44 (above were BRCA1-associated hub genes) and ITGAX (BRCA2-associated hub genes) expression were more obviously negatively correlated with tumor purity in Basal-like BC, with an undefined correlation for the tumor purity of luminalsubtype. BUB1, CCNB1, BUB1B, CCNA2, KIF11, CDC20, TTK, NCAPG, influenced by BRCA1/2 mutations, which were closely related to regulation of cell cycle and DNA repair, showed more obviously positive

correlation with tumor purity in luminal-type BC. In other words, other types of BC would weaken the significant correlation between tumor purity of basal-like subtype or luminal subtype and these above hub genes. For LIPE and FABP4, BRCA2-associated hub genes closely related to biological functions including metabolic processes, inflammatory response and cell migration, our study found that their expression levels were significantly negatively correlated with the tumor purity of luminal subtype; by comparison, were significantly positively correlated with infiltrating immune cells, CD4+/CD8+ T cells and dendritic cells. These results suggested that could recruit immune cells in the tumor microenvironment (TME) in BRCA1/2-mutant BC, especially on CD4+/CD8+ T cells, neutrophils and dendritic cells.

Figure S1

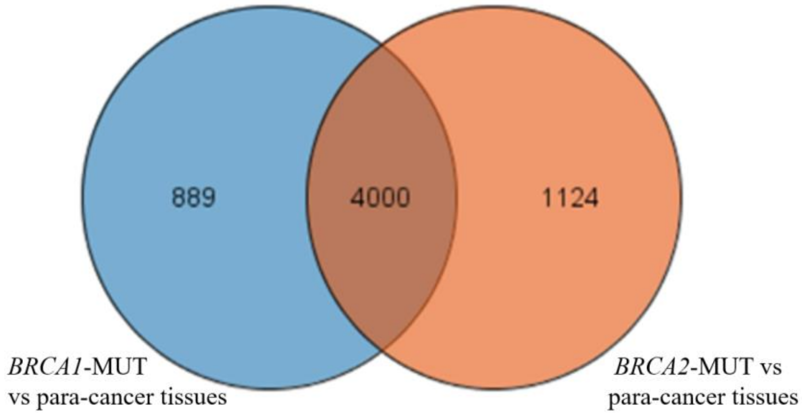

Figure S2

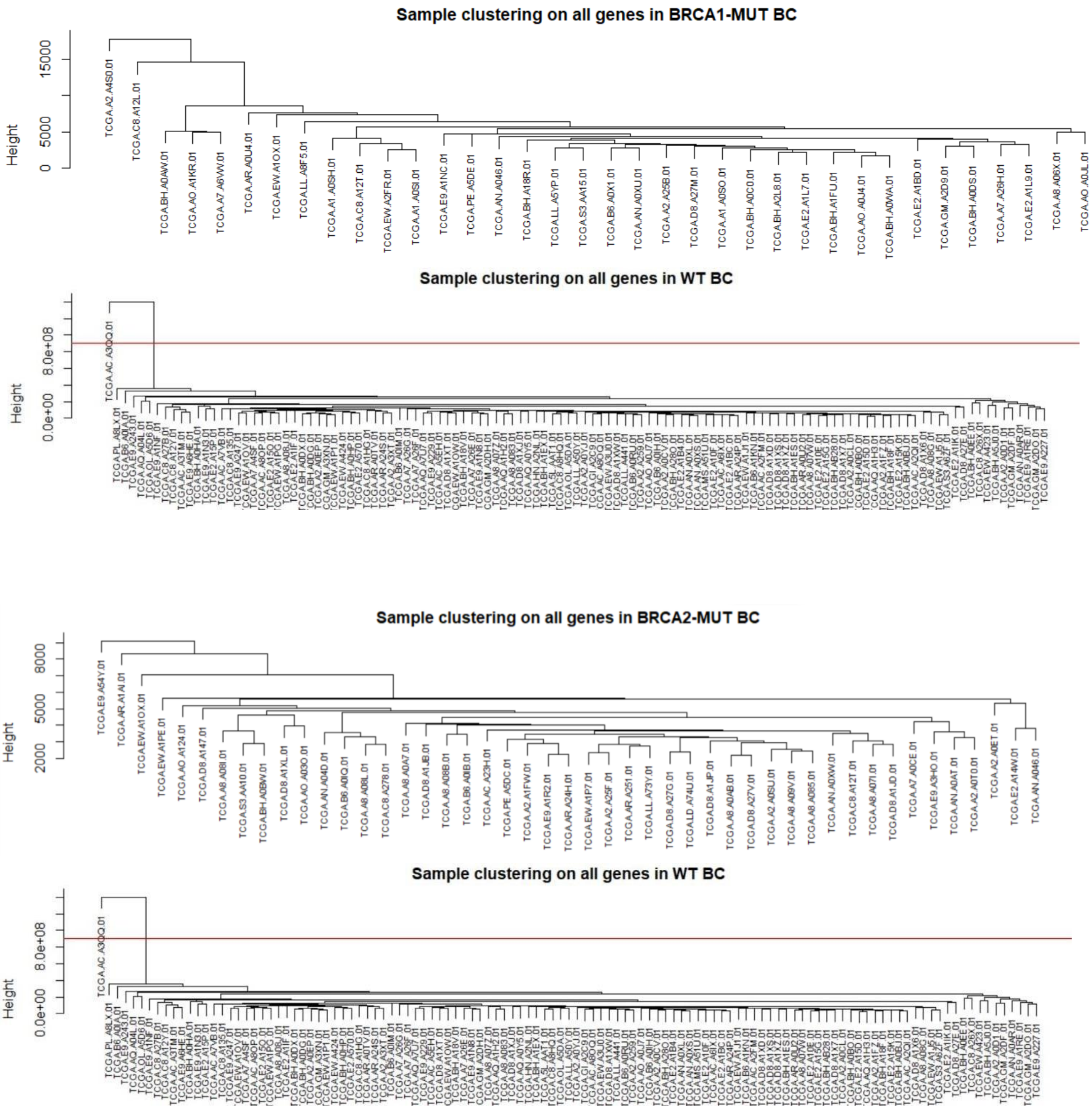

Sample clustering on all genes in BRCA1-MUT BC
Height
0
5000
15000
Sample clustering on all genes in WT BC
Height
0.0e+00
8.0e+08
Sample clustering on all genes in BRCA2-MUT BC
Height
2000
5000
8000
Sample clustering on all genes in WT BC
Height
0.0e+00
8.0e+08

Figure S3

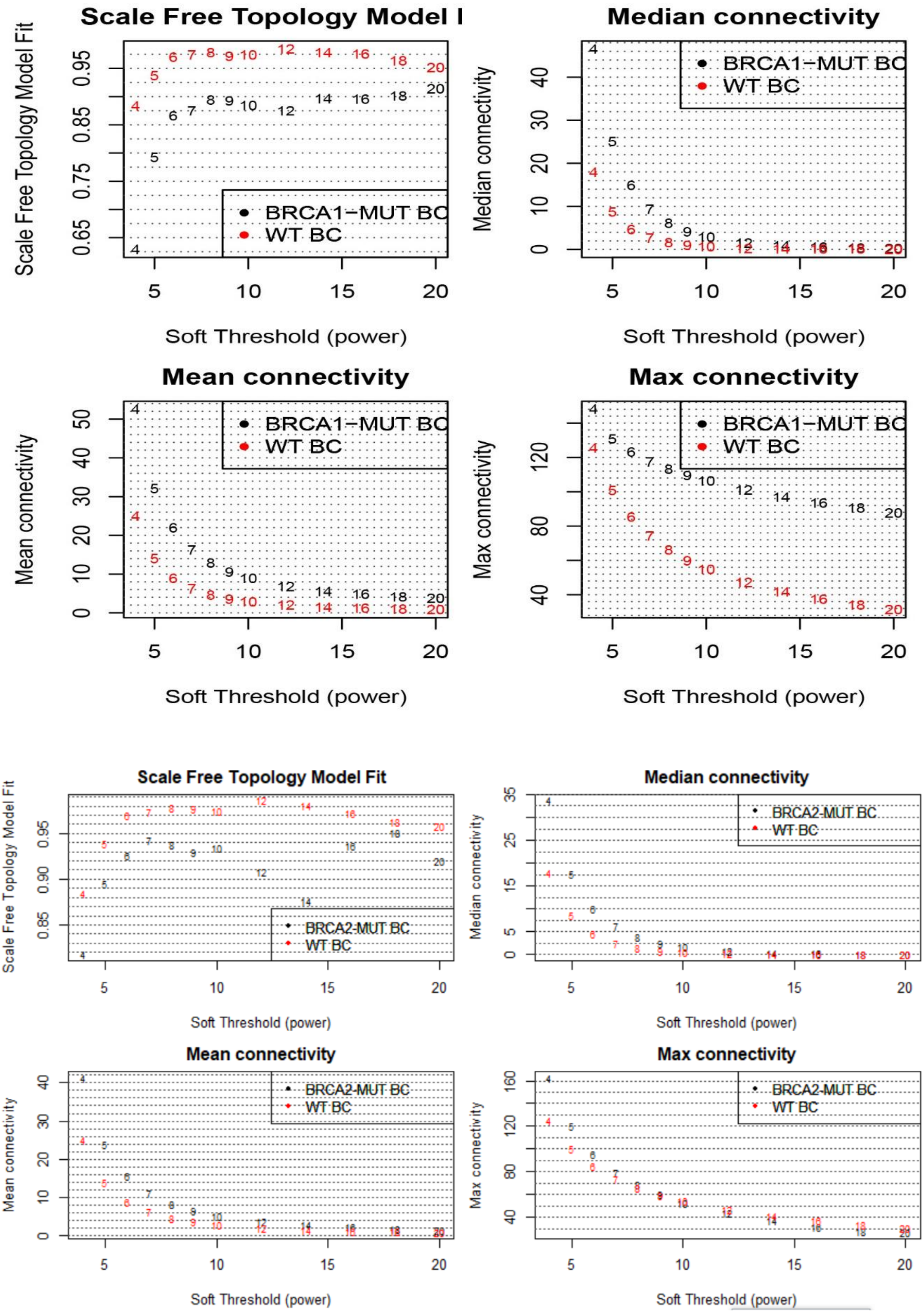
Scale Free Topology Model I
Scale Free Topology Model Fit
Soft Threshold (power)
BRCA1−MUT BC
WT BC
Median connectivity
Mean connectivity
Max connectivity
Scale Free Topology Model Fit
BRCA2-MUT BC
WT BC
Median connectivity
Mean connectivity
Max connectivity

Figure S4

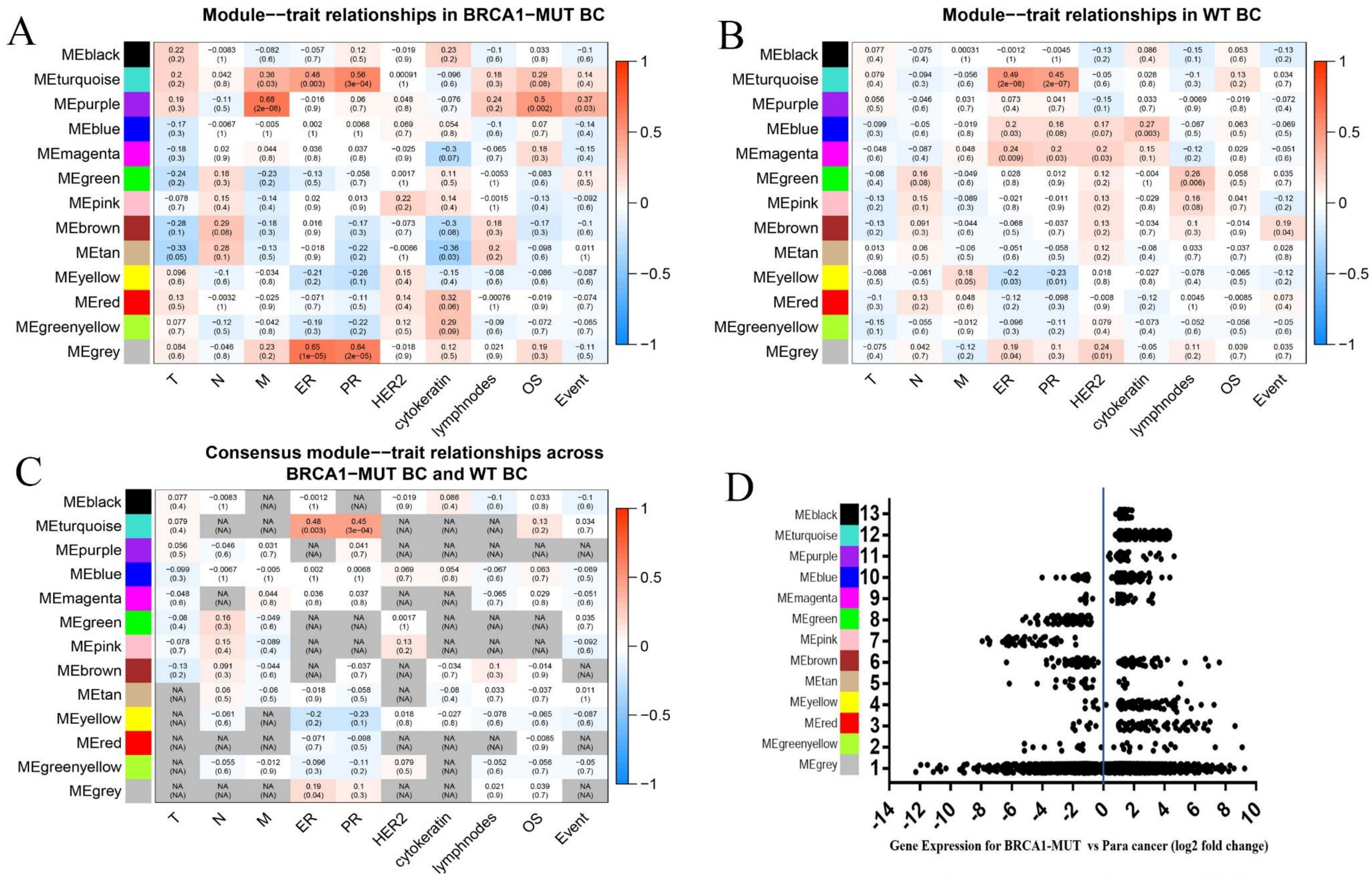

A
Module−−trait relationships in BRCA1−MUT BC
B
Module−−trait relationships in WT BC
C
Consensus module−−trait relationships across BRCA1−MUT BC and WT BC
MEblack
MEturquoise
MEpurple
MEblue
MEmagenta
MEgreen
MEpink
MEbrown
MEtan
MEyellow
MEred
MEgreenyellow
MEgrey
T
N
M
ER
PR
HER2
cytokeratin
lymphnodes
OS
Event
1
0.5
0
−0.5
−1
D
13
12
11
10
9
8
7
6
5
4
3
2
1
-14 -12 -10 -8 -6 -4 -2 0 2 4 6 8 10
Gene Expression for BRCA1-MUT vs Para cancer (log2 fold change)

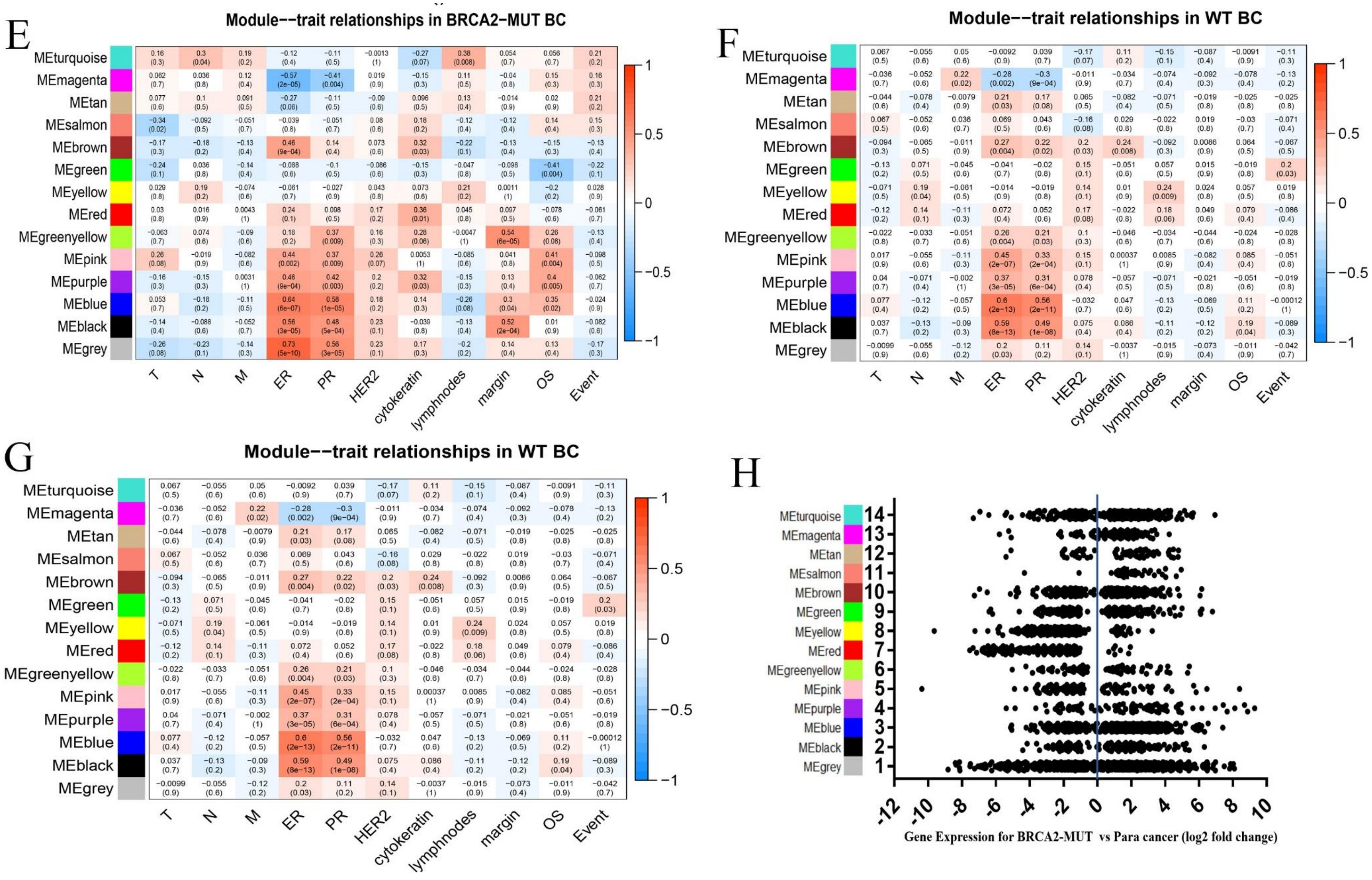
E
Module--trait relationships in BRCA2-MUT BC
MEturquoise
MEmagenta
MEtan
MEsalmon
MEbrown
MEgreen
MEyellow
MEred
MEgreenyellow
MEpink
MEpurple
MEblue
MEblack
MEgrey
T
N
M
ER
PR
HER2
cytokeratin
lymphnodes
margin
OS
Event
1
0.5
0
−0.5
−1
F
Module--trait relationships in WT BC
G
Module--trait relationships in WT BC
H
14
13
12
11
10
9
8
7
6
5
4
3
2
1
-12
-10
-8
-6
-4
-2
0
2
4
6
8
10
Gene Expression for BRCA2-MUT vs Para cancer (log2 fold change)

Figure S5

The ordinate is the subtypes of breast cancer (BC-all, BC-Basal, BC-luminal, BC-Her2) and the abscissa represents different analysis indexes of infiltration level, in order as follows: tumor purity, B cells, CD8+ T cells, CD4+ T cells, macrophages, neutrophils, and dendritic cells.

A) Correlation of ISG15 expression with immune infiltration level in breast cancer.

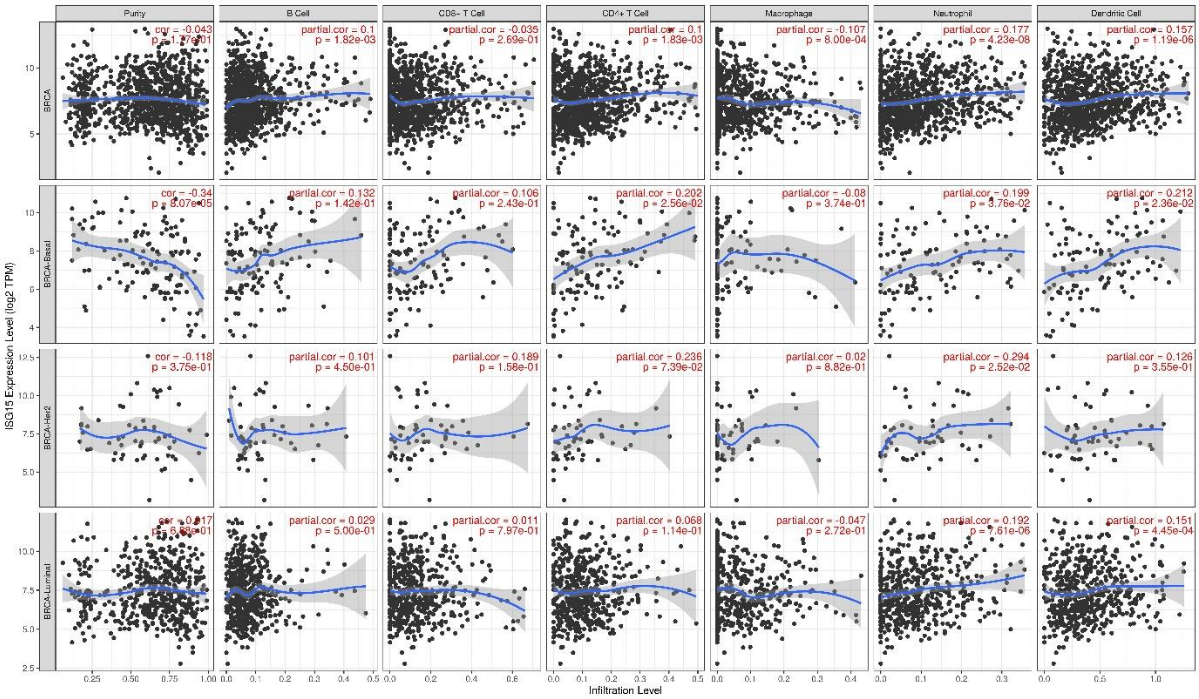

B) Correlation of IFIT1 expression with immune infiltration level in breast cancer.

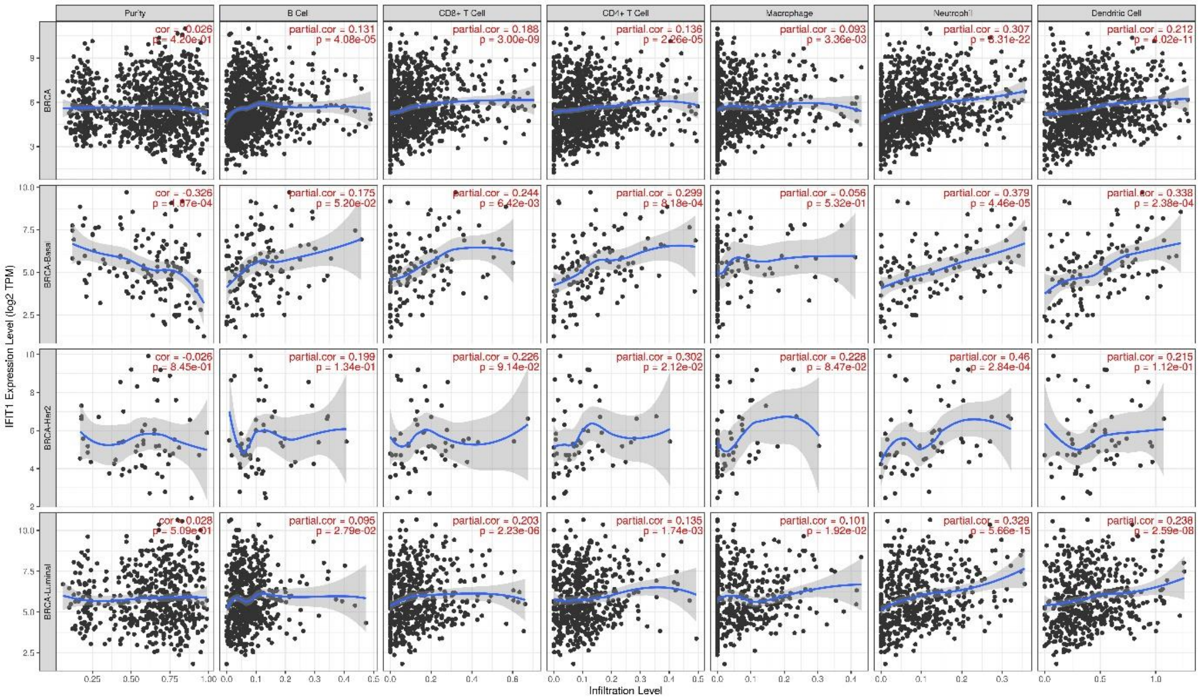

C) Correlation of MX1 expression with immune infiltration level in breast cancer.

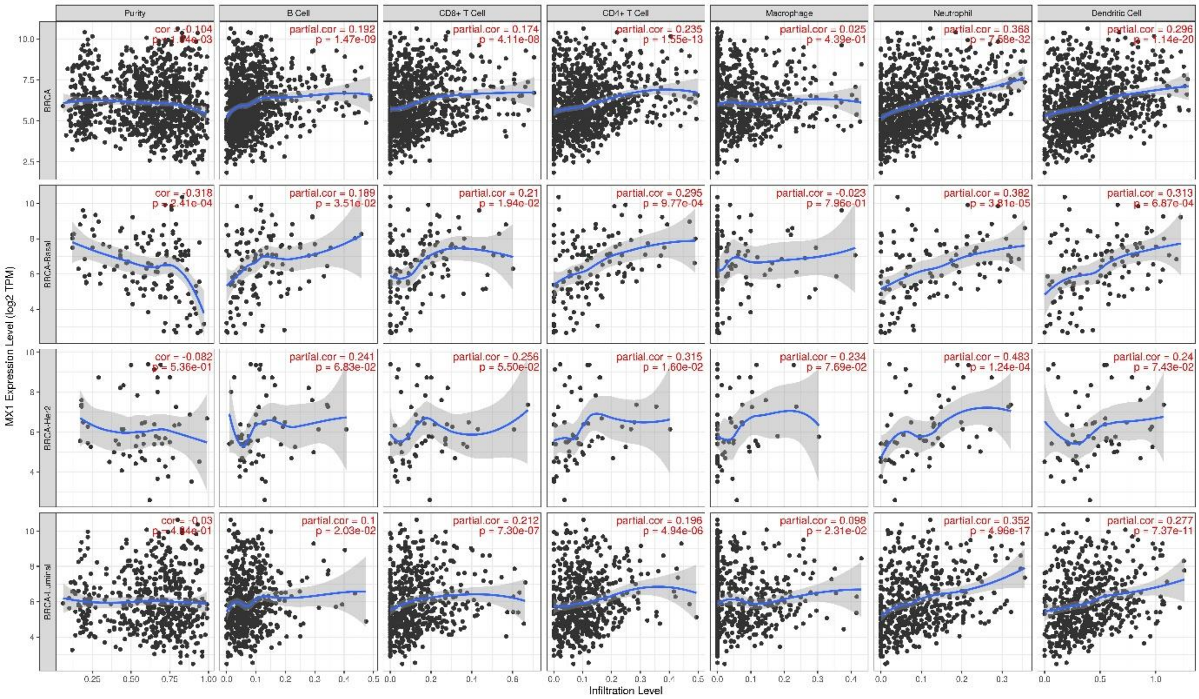

D) Correlation of DDX58 expression with immune infiltration level in breast cancer.

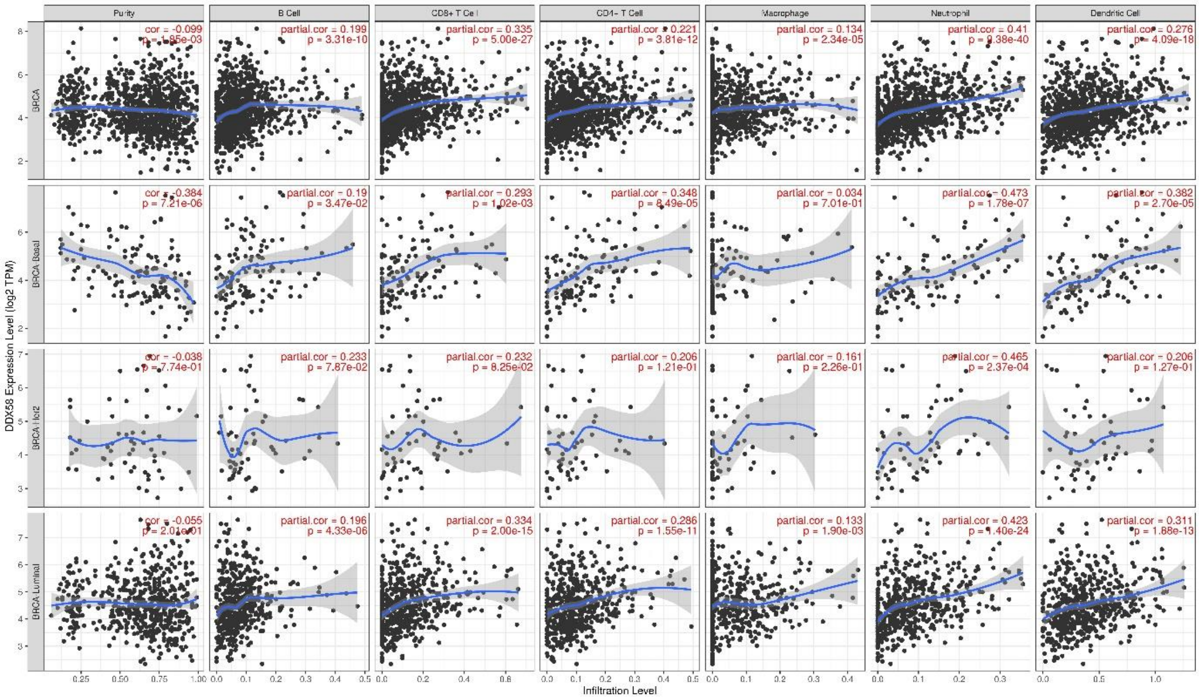

E) Correlation of STAT1 expression with immune infiltration level in breast cancer.

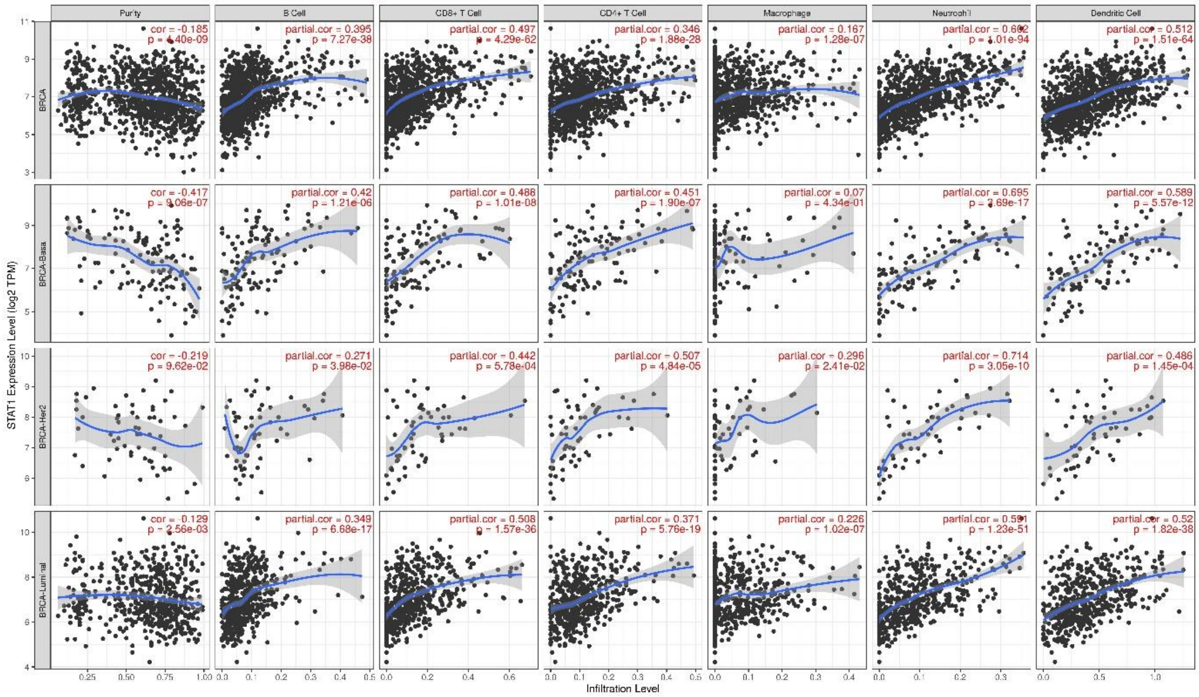

F) Correlation of RASD2 expression with immune infiltration level in breast cancer.

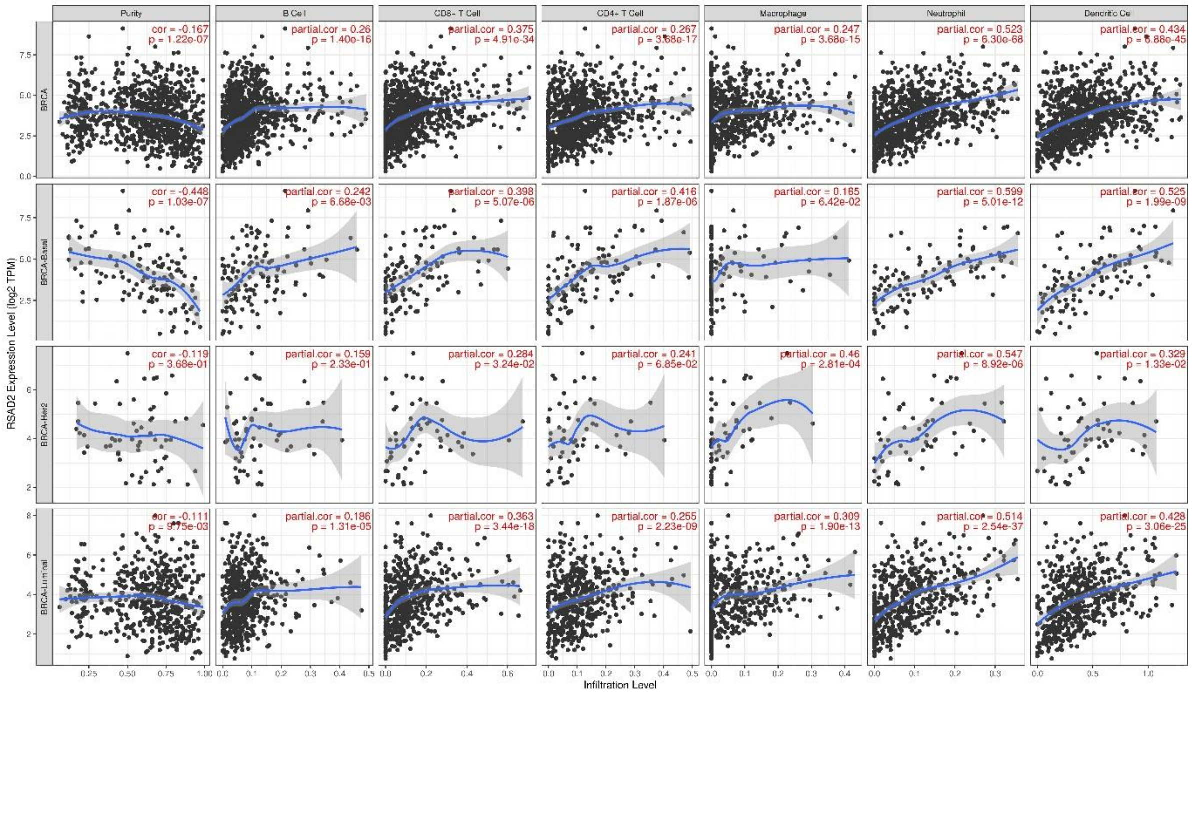

G) Correlation of OAS1 expression with immune infiltration level in breast cancer.

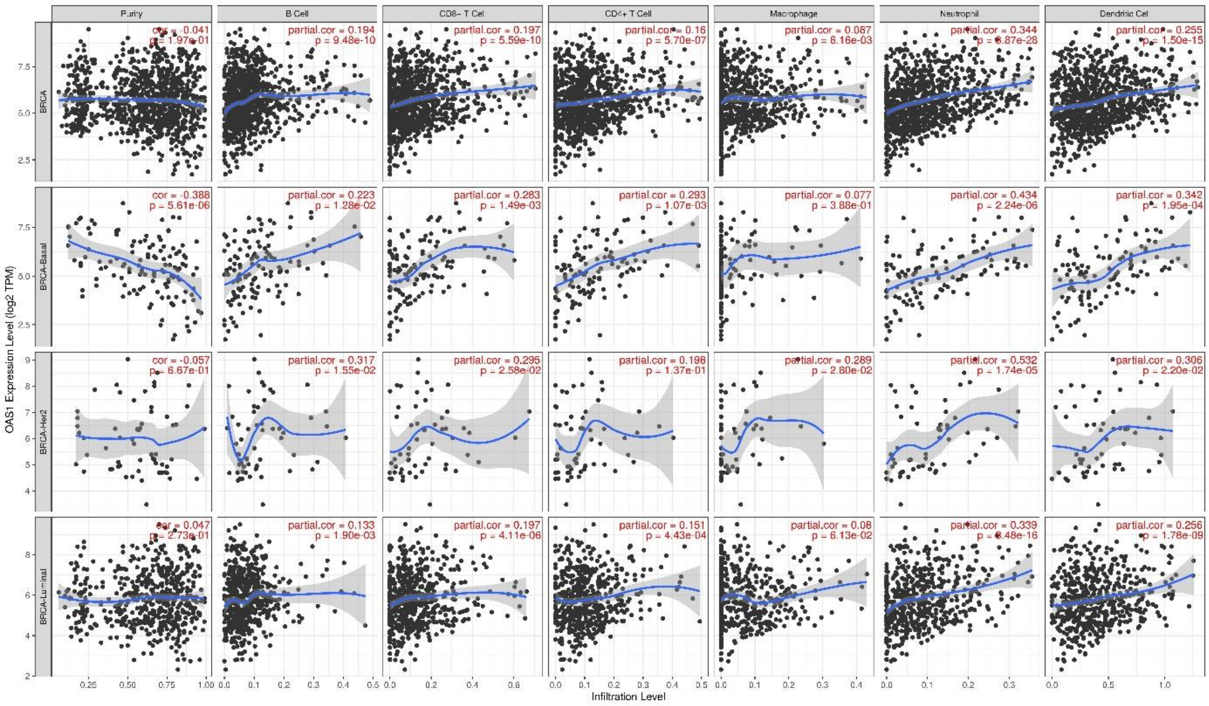

H) Correlation of IFI44 expression with immune infiltration level in breast cancer.

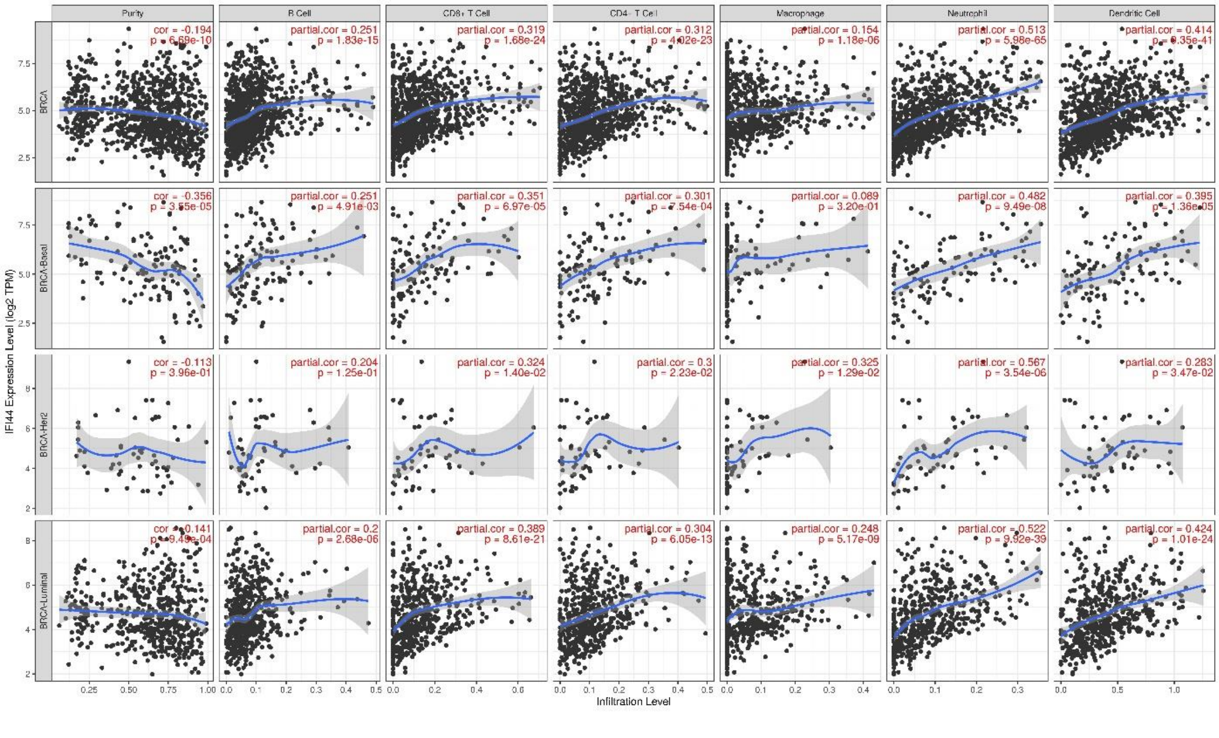

I) Correlation of ITGAX expression with immune infiltration level in breast cancer.

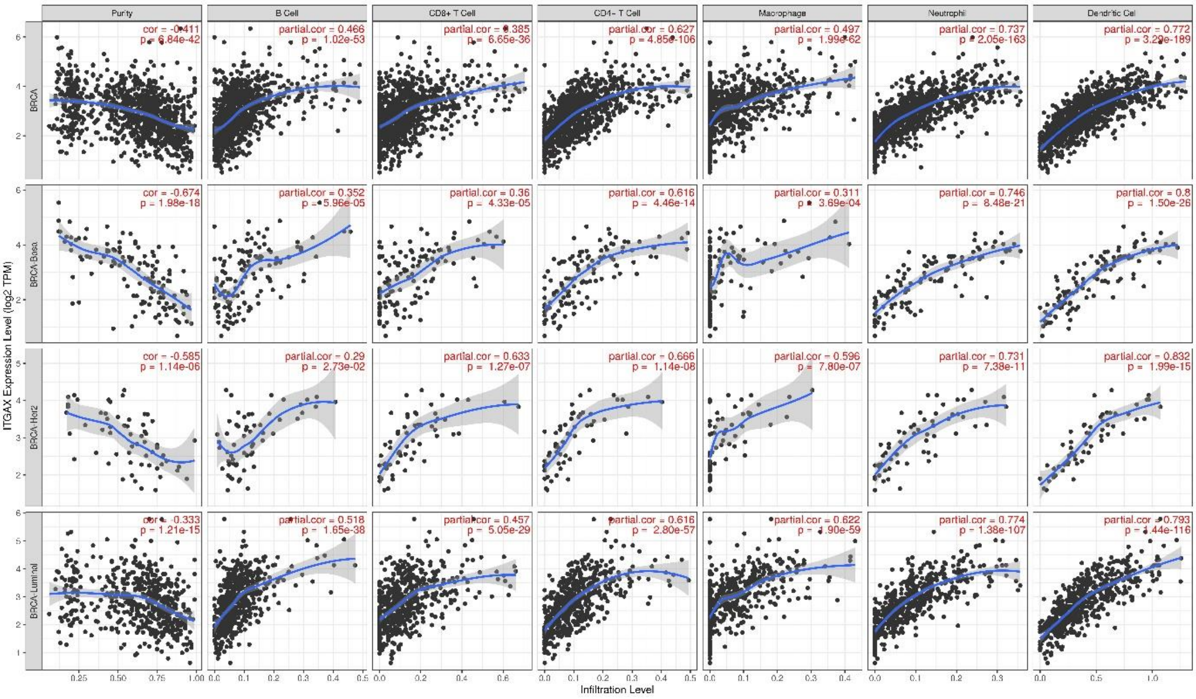

J) Correlation of BUB1 expression with immune infiltration level in breast cancer.

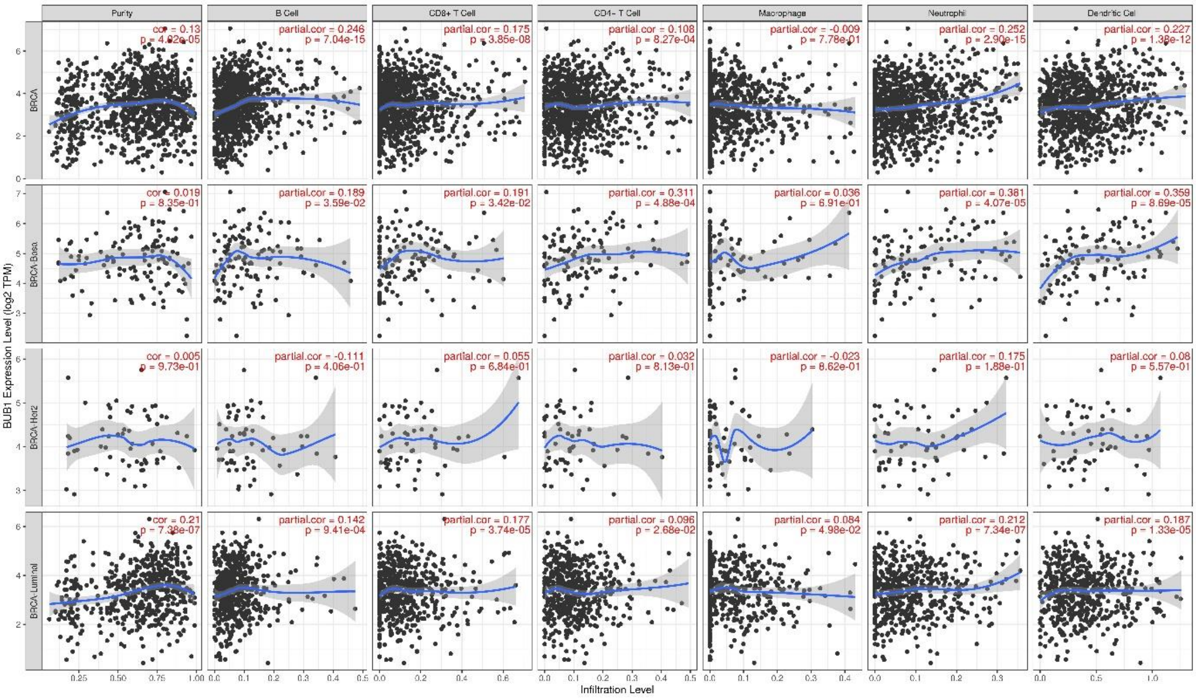

K) Correlation of CCNB1 expression with immune infiltration level in breast cancer.

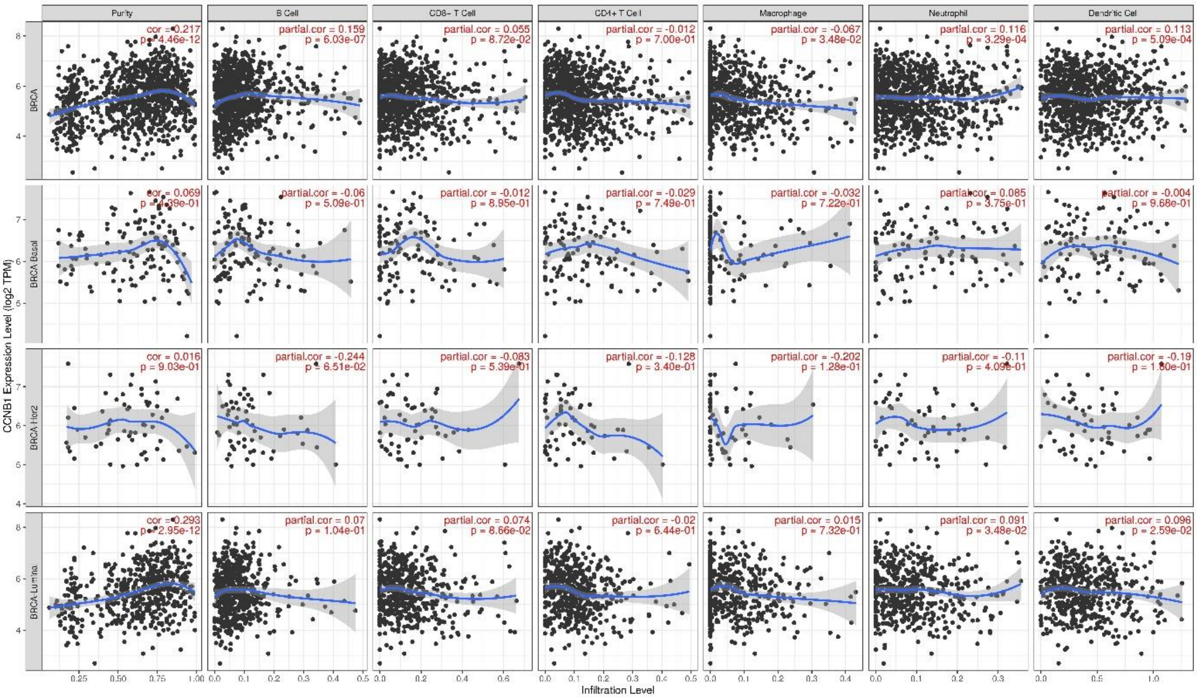

L) Correlation of BUB1B expression with immune infiltration level in breast cancer.

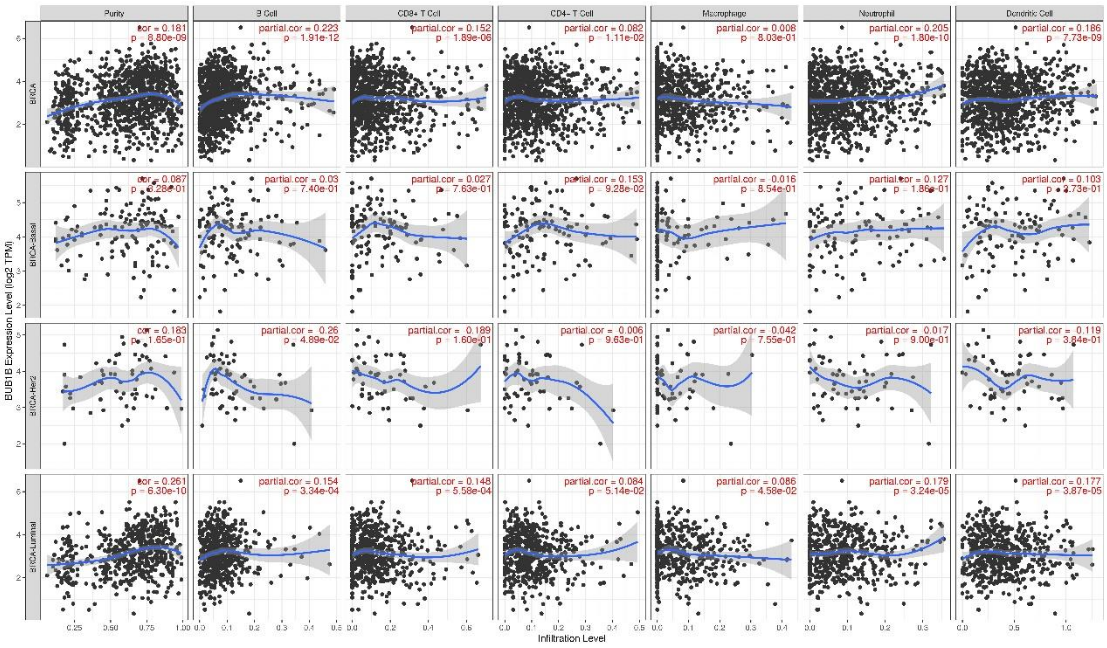

M) Correlation of CCNA2 expression with immune infiltration level in breast cancer.

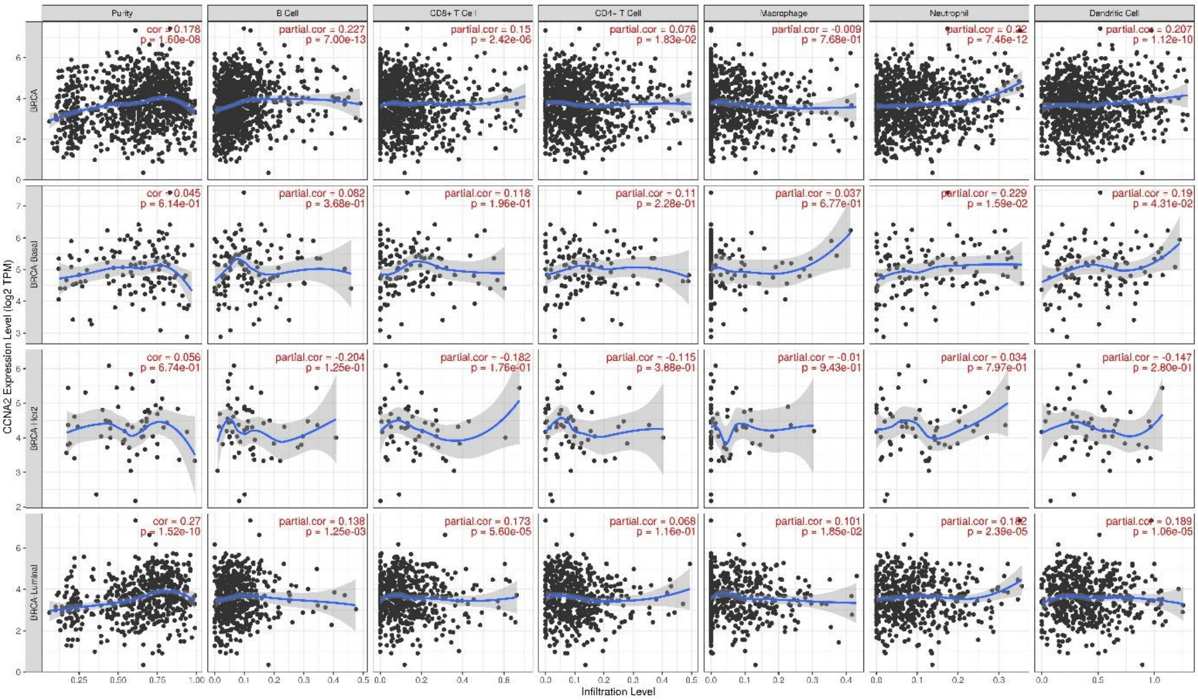

N) Correlation of KIF11 expression with immune infiltration level in breast cancer.

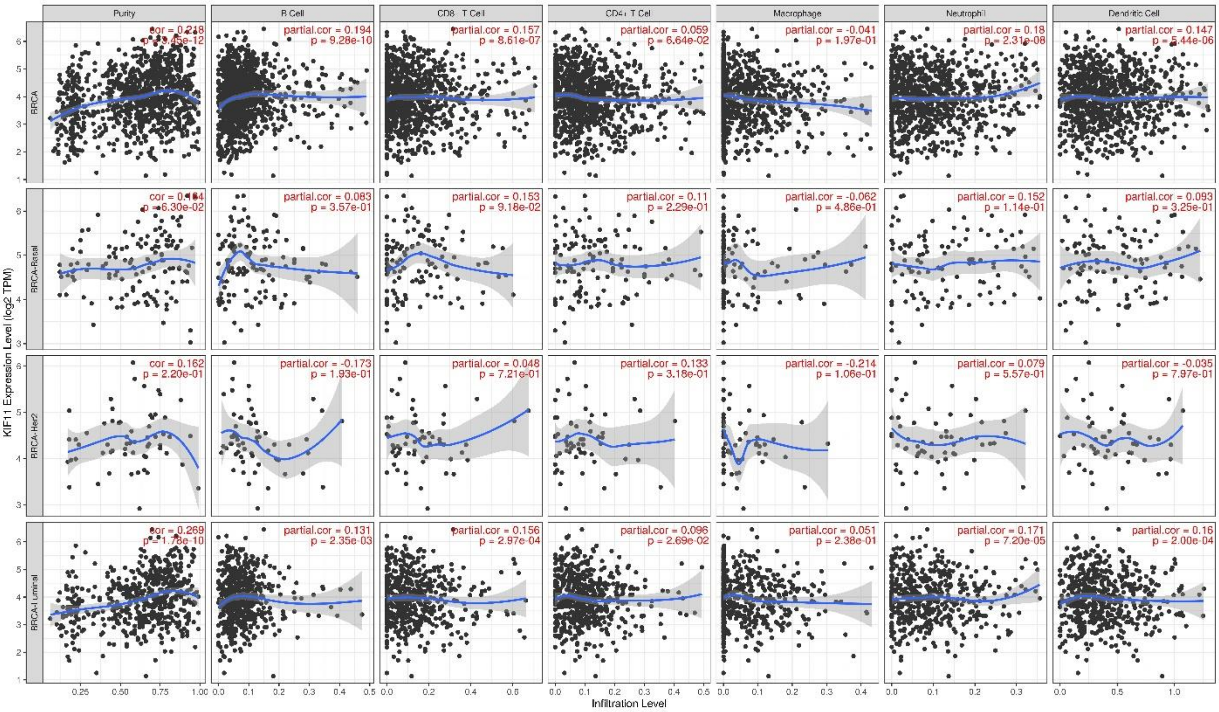

O) Correlation of CDC20 expression with immune infiltration level in breast cancer.

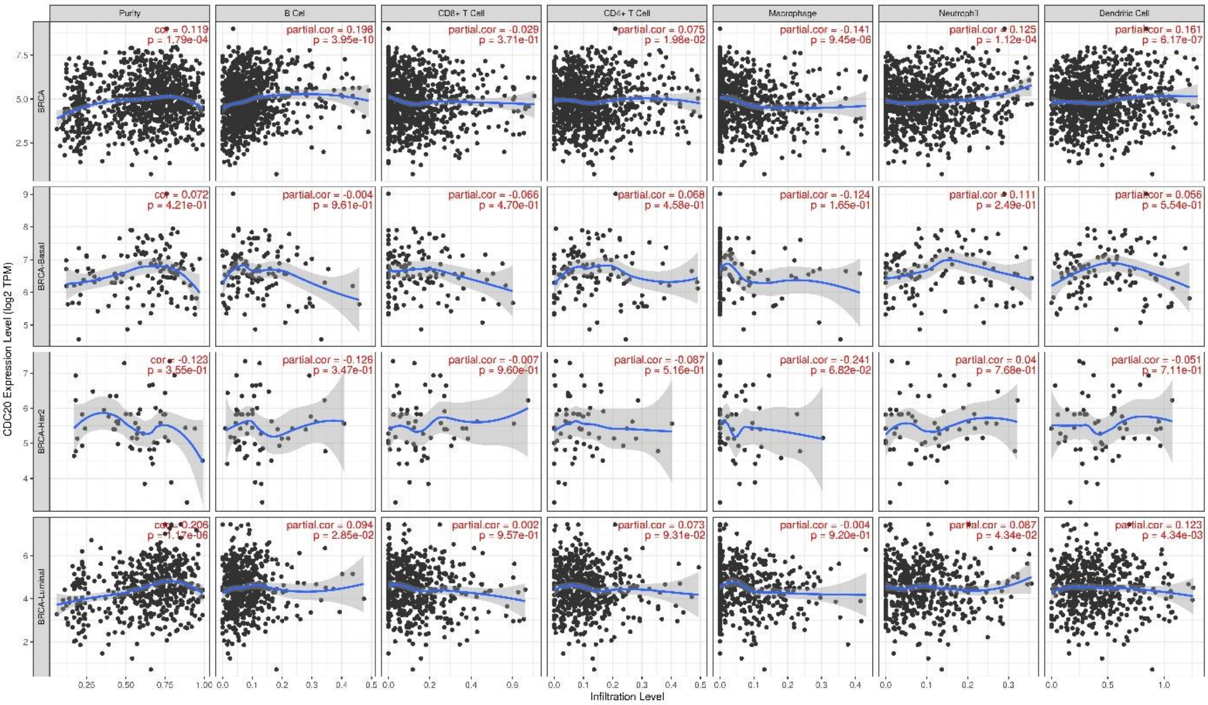

P) Correlation of TTK expression with immune infiltration level in breast cancer.

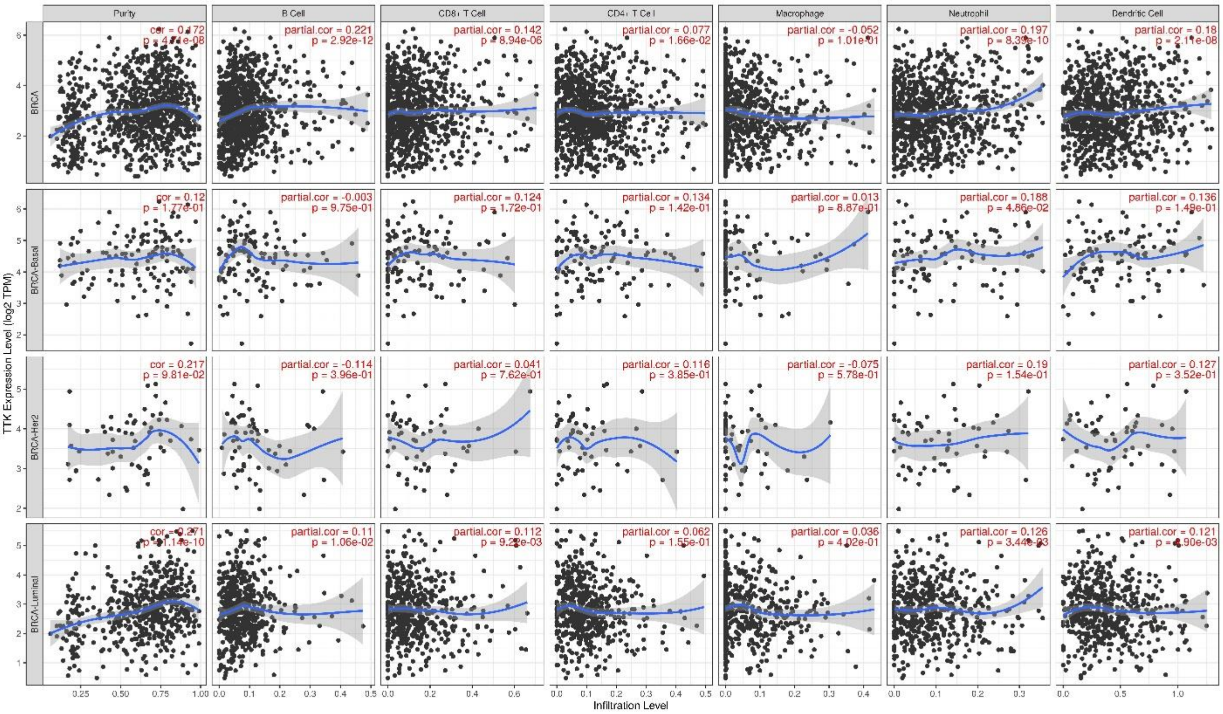

Q) Correlation of NCAPG expression with immune infiltration level in breast cancer.

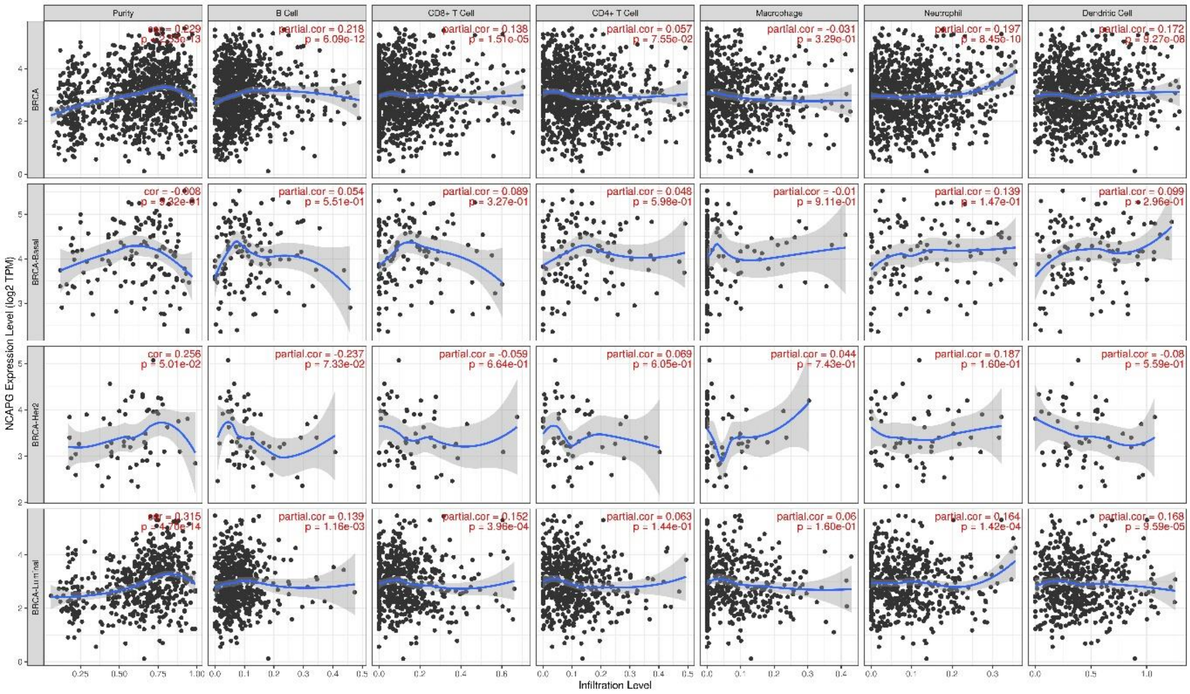

R) Correlation of LIPE expression with immune infiltration level in breast cancer.

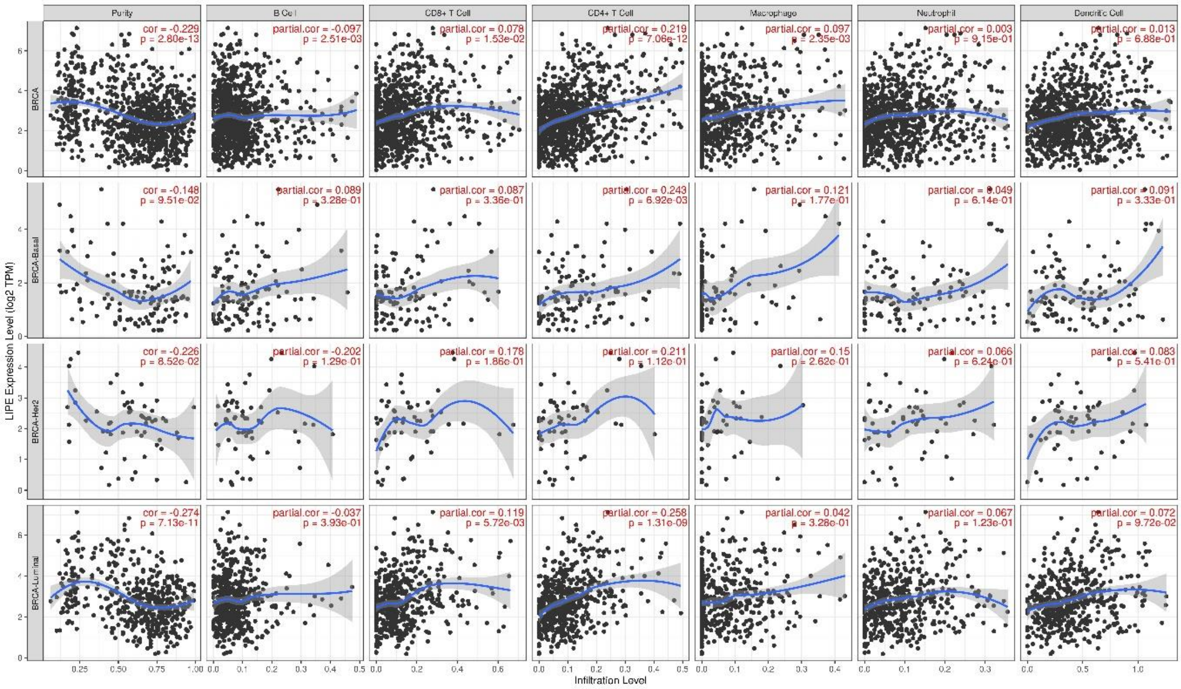

S) Correlation of FABP4 expression with immune infiltration level in breast cancer.

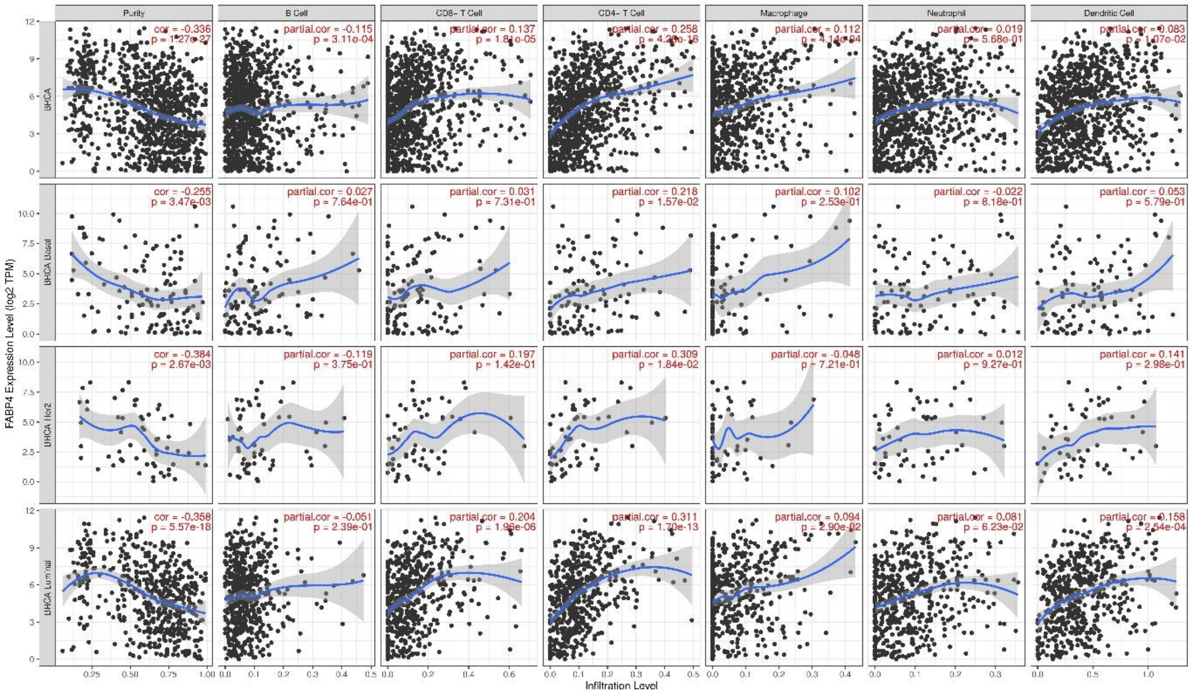

Supplement: Supplementary file 1 [file Data_Sheet_1.pdf]
