# Supplementary material for: Comprehensive Analysis of Regulatory Factors and Immune-Associated Patterns to Decipher Common and BRCA1/2 Mutation-Type-Specific Critical Regulation in Breast Cancer
Source: Front Cell Dev Biol. 2021 Oct 18;9:750897. doi: 10.3389/fcell.2021.750897 (PMC8558486; doi:10.3389/fcell.2021.750897)
Supplement: Supplementary file 4 [file Table_3.DOCX]

**Table S2. Correlation analysis between related hub genes and markers of infiltrating immune cell in TIMER.**

**A) Correlation analysis between related hub genes and markers of infiltrating immune monocyte/macrophage in TIMER.**

| Gene | Gene markers | | | | | | | | | | | |
| --- | --- | --- | --- | --- | --- | --- | --- | --- | --- | --- | --- | --- |
|  | Monocyte | | | TAM (tumor-associated macrophage) | | | M1 macrophage | | | M2 macrophage | | |
|  | CD14 | CD86 | CD16 | CD68 | CCL2 | CCL5 | INOS | CXCL10 | TNF-a | CD206 | CD163 | IL10 |
| ISG15 | 0.253*** | 0.295*** | 0.225*** | 0.255*** | 0.258*** | 0.347*** | -0.101** | 0.461*** | 0.136*** | -0.048 | 0.235*** | 0.289*** |
| MX1 | 0.251*** | 0.436*** | 0.337*** | 0.339*** | 0.385*** | 0.431*** | -0.035 | 0.596*** | 0.259*** | 0.109** | 0.364*** | 0.432*** |
| IFIT1 | 0.152*** | 0.405*** | 0.348*** | 0.312*** | 0.329*** | 0.306*** | -0.041 | 0.515*** | 0.171*** | 0.08* | 0.08* | 0.402*** |
| DDX58 | 0.073* | 0.421*** | 0.358*** | 0.294*** | 0.327*** | 0.3*** | 0.005 | 0.513*** | 0.228*** | 0.166*** | 0.166*** | 0.384*** |
| STAT1 | 0.217*** | 0.631*** | 0.558*** | 0.519*** | 0.426*** | 0.552*** | -0.016 | 0.789*** | 0.298*** | 0.36*** | 0.36*** | 0.585*** |
| RSAD2 | 0.243*** | 0.599*** | 0.568*** | 0.504*** | 0.464*** | 0.437*** | 0.006 | 0.698*** | 0.276*** | 0.231*** | 0.502*** | 0.561*** |
| OAS1 | 0.204*** | 0.446*** | 0.381*** | 0.363*** | 0.314*** | 0.34*** | -0.066* | 0.538*** | 0.19*** | 0.101** | 0.352*** | 0.428*** |
| IFI44 | 0.306*** | 0.547*** | 0.432*** | 0.424*** | 0.46*** | 0.492*** | 0.035 | 0.679*** | 0.3*** | 0.24*** | 0.461*** | 0.534*** |
| ITGAX | 0.543*** | 0.805*** | 0.679*** | 0.823*** | 0.424*** | 0.447*** | 0.145** | 0.482*** | 0.362*** | 0.488*** | 0.681*** | 0.634*** |
| BUB1 | 0.028 | 0.287*** | 0.277*** | 0.258*** | 0.222*** | 0.255*** | 0.101** | 0.483*** | 0.26*** | 0.228*** | 0.347*** | 0.332*** |
| CCNB1 | -0.014 | 0.295*** | 0.203*** | 0.178*** | 0.134*** | 0.181*** | -0.016 | 0.403*** | 0.202*** | 0.104** | 0.237*** | 0.248*** |
| BUB1B | -0.015 | 0.246*** | 0.251*** | 0.221*** | 0.176*** | 0.182*** | 0.101** | 0.414*** | 0.219*** | 0.206*** | 0.306*** | 0.297*** |
| CCNA2 | 0.062 | 0.275*** | 0.261*** | 0.249*** | 0.216*** | 0.256*** | 0.07* | 0.47*** | 0.246*** | 0.22*** | 0.335*** | 0.327*** |
| KIF11 | -0.017 | 0.224*** | 0.217*** | 0.176*** | 0.18*** | 0.211*** | 0.072* | 0.413*** | 0.252*** | 0.143*** | 0.245*** | 0.259*** |
| CDC20 | 0.148*** | 0.175*** | 0.145*** | 0.18*** | 0.174*** | 0.277*** | 0.077* | 0.41*** | 0.25*** | 0.097** | 0.241*** | 0.211*** |
| TTK | 0.001 | 0.233*** | 0.221*** | 0.18*** | 0.203*** | 0.241*** | 0.092** | 0.468*** | 0.282*** | 0.182*** | 0.273*** | 0.271*** |
| NCAPG | 0.03 | 0.265*** | 0.241*** | 0.221*** | 0.211*** | 0.246*** | 0.04 | 0.466*** | 0.256*** | 0.188*** | 0.303*** | 0.309*** |
| LIPE | -0.017 | -0.168** | -0.125** | -0.036 | -0.055 | -0.048 | -0.229** | -0.267*** | -0.128*** | 0.124*** | -0.026* | -0.048* |
| FABP4 | -0.031 | -0.042 | -0.131** | 0.026 | 0.069* | 0.053 | 0.119*** | -0.202*** | 0.225*** | 0.199*** | 0.029 | 0.042* |

In Timer dataset, the expression scatterplots of two genes adjusted by tumor purity in breast cancer (1093 cases), together with the Spearman’s rho value and estimated statistical significance, could reflect the correlation between our hub genes and gene markers of infiltrating immune cell. *p<0.05, **p<0.01, *** p<0.001.

**B) Correlation analysis between related hub genes and gene markers of infiltrating immune neutrophils/natural killer cell/dendritic cell in TIMER.**

| Gene | Gene markers | | | | | | | | | | | |
| --- | --- | --- | --- | --- | --- | --- | --- | --- | --- | --- | --- | --- |
|  | Neutrophils | | | natural killer cell | | | | Dendritic cell | | | | |
|  | CD11b | CCR7 | CD15 | KIR2DL1 | KIR2DL3 | KIR2DL4 | KIR3DL1 | HLA-DQB1 | HLA-DPB1 | HLA-DRA | BDCA-1  （CD1C） | BDCA-4(NRP1) |
| ISG15 | 0.068* | 0.207*** | -0.001 | 0.142*** | 0.207*** | 0.386*** | 0.169*** | 0.135*** | 0.132*** | 0.107*** | -0.131*** | -0.237*** |
| MX1 | 0.181*** | 0.301*** | 0.22*** | 0.19*** | 0.254*** | 0.471*** | 0.232*** | 0.158*** | 0.15*** | 0.245*** | -0.027* | -0.05* |
| IFIT1 | 0.156*** | 0.209*** | 0.121*** | 0.124*** | 0.214*** | 0.375*** | 0.167*** | 0.089** | 0.075** | 0.195*** | 0.066* | 0.006 |
| DDX58 | 0.259*** | 0.26*** | 0.236*** | 0.162*** | 0.23*** | 0.362*** | 0.195*** | 0.11*** | 0.082** | 0.275*** | 0.046* | 0.169*** |
| STAT1 | 0.339*** | 0.431*** | 0.367*** | 0.294*** | 0.406*** | 0.589*** | 0.361*** | 0.306*** | 0.289*** | 0.516*** | 0.065* | 0.113*** |
| RSAD2 | 0.304*** | 0.27*** | 0.345*** | 0.208*** | 0.298*** | 0.471*** | 0.28*** | 0.193*** | 0.197*** | 0379*** | -0.062* | 0.167** |
| OAS1 | 0.206*** | 0.248*** | 0.138*** | 0.143*** | 0.251*** | 0.404*** | 0.192*** | 0.142*** | 0.144*** | 0.265*** | -0.055* | -0.029* |
| IFI44 | 0.253*** | 0.321*** | 0.339*** | 0.229*** | 0.306*** | 0.523*** | 0.287*** | 0.219*** | 0.222*** | 0.357*** | 0.016 | 0.054* |
| ITGAX | 0.693*** | 0.424*** | 0.365*** | 0.246*** | 0.264*** | 0.313*** | 0.309*** | 0.486*** | 0.641*** | 0.71*** | 0.287*** | 0.273*** |
| BUB1 | 0.113*** | 0.202*** | 0.301*** | 0.182*** | 0.231*** | 0.321*** | 0.214*** | 0.061* | -0.063* | 0.152*** | -0.113*** | 0.021 |
| CCNB1 | 0.038 | 0.128*** | 0.129*** | 0.121*** | 0.181*** | 0.262*** | 0.128*** | 0.039* | -0.105*** | 0.079* | -0.159*** | -0.093* |
| BUB1B | 0.089** | 0.18*** | 0.256*** | 0.144*** | 0.197*** | 0.264*** | 0.174*** | 0.04* | -0.089** | 0.125*** | -0.11*** | 0.054* |
| CCNA2 | 0.091** | 0.186*** | 0.258*** | 0.175*** | 0.225*** | 0.317*** | 0.222*** | 0.068* | -0.05* | 0.146*** | -0.118*** | -0.017 |
| KIF11 | 0.081* | 0.164*** | 0.201*** | 0.141*** | 0.199*** | 0.267*** | 0.181*** | 0.031* | -0.094** | 0.098** | -0.108*** | -0.006 |
| CDC20 | 0.021 | 0.165*** | 0.198*** | 0.191*** | 0.184*** | 0.29*** | 0.19*** | 0.092** | -0.016 | 0.067* | -0.15*** | -0.162*** |
| TTK | 0.077* | 0.176*** | 0.29*** | 0.168*** | 0.211*** | 0.304*** | 0.189*** | 0.063* | -0.105*** | 0.124*** | -0.121*** | 0.001 |
| NCAPG | 0.074* | 0.174*** | 0.225*** | 0.174*** | 0.231*** | 0.318*** | 0.212*** | 0.057* | -0.089** | 0.123*** | -0.15*** | -0.037* |
| LIPE | 0.065* | 0.151*** | -0.054 | -0.115** | -0.104** | -0.204*** | -0.052 | -0.007 | 0.155*** | 0.008 | 0.308*** | 0.201*** |
| FABP4 | 0.006 | 0.24*** | 0.02 | -0.058 | -0.104*** | -0.167*** | -0.01 | -0.015 | 0.118* | 0.012 | 0.399*** | 0.227*** |

**C) Correlation analysis between related hub genes and gene markers of adaptive immunity cells in TIMER.**

| Gene | Gene markers | | | | | | | | | | | | | |
| --- | --- | --- | --- | --- | --- | --- | --- | --- | --- | --- | --- | --- | --- | --- |
|  | CD8^+^ T cell | | T cell (general) | | | B cell | | | | T cell exhaustion | | | | |
|  | CD8A | CD8B | CD3D | CD3E | CD2 | CD19 | CD20  (MS4A1) | CD138  (SDC1) | CD23  (FCER2) | PD-1  (PDCD1) | CTLA4 | LAG3 | TIM-3  (HAVCR2) | GZMB |
| ISG15 | 0.178*** | 0.171*** | 0.234*** | 0.198*** | 0.235*** | 0.121*** | 0.032* | 0.017 | 0.019 | 0.299*** | 0.339*** | 0.522*** | 0.232*** | 0.333*** |
| MX1 | 0.305*** | 0.275*** | 0.307*** | 0.309*** | 0.364*** | 0.156*** | 0.142*** | 0.021 | 0.036* | 0.32*** | 0.431*** | 0.555*** | 0.363*** | 0.412*** |
| IFIT1 | 0.23*** | 0.141*** | 0.192*** | 0.207*** | 0.27*** | 0.046* | 0.066* | -0.02 | -0.043* | 0.181*** | 0.31*** | 0.417*** | 0.358*** | 0.263*** |
| DDX58 | 0.291*** | 0.173*** | 0.206*** | 0.247*** | 0.312*** | 0.084** | 0.167*** | -0.023* | 0.006 | 0.154*** | 0.303*** | 0.342*** | 0.372*** | 0.246*** |
| STAT1 | 0.523*** | 0.396*** | 0.478*** | 0.505*** | 0.586*** | 0.277*** | 0.319*** | 0.033* | 0.082** | 0.46*** | 0.616*** | 0.596*** | 0.55*** | 0.557*** |
| RSAD2 | 0.34*** | 0.268*** | 0.318*** | 0.332*** | 0.416*** | 0.129*** | 0.143*** | 0.194 | -0.017 | 0.298*** | 0.483*** | 0.534*** | 0.567*** | 0.418*** |
| OAS1 | 0.256*** | 0.174*** | 0.243*** | 0.258*** | 0.319*** | 0.1** | 0.105*** | -0.006 | 0.015 | 0.242*** | 0.374*** | 0.464*** | 0.395*** | 0.317*** |
| IFI44 | 0.374*** | 0.318*** | 0.379*** | 0.374*** | 0.443*** | 0.21*** | 0.206*** | 0.037* | 0.084** | 0.39*** | 0.544*** | 0.608*** | 0.46*** | 0.492*** |
| ITGAX | 0.44*** | 0.345*** | 0.494*** | 0.511*** | 0.543*** | 0.338*** | 0.358*** | 0.136*** | 0.27*** | 0.469*** | 0.57*** | 0.445*** | 0.837*** | 0.409*** |
| BUB1 | 0.216*** | 0.185*** | 0.188*** | 0.207*** | 0.266*** | 0.157*** | 0.148*** | 0.132*** | 0.001 | 0.229*** | 0.384*** | 0.342*** | 0.245*** | 0.365*** |
| CCNB1 | 0.129*** | 0.105*** | 0.132*** | 0.137*** | 0.191*** | 0.87** | 0.065* | 0.116*** | -0.064* | 0.157*** | 0.306*** | 0.31*** | 0.17*** | 0.291*** |
| BUB1B | 0.179*** | 0.125*** | 0.148*** | 0.174*** | 0.223*** | 0.118*** | 0.13*** | 0.093** | -0.009 | 0.172*** | 0.329*** | 0.267*** | 0.221*** | 0.297*** |
| CCNA2 | 0.205*** | 0.182*** | 0.199*** | 0.206*** | 0.264*** | 0.163*** | 0.149*** | 0.118*** | 0.011 | 0.242*** | 0.392*** | 0.347*** | 0.238*** | 0.384*** |
| KIF11 | 0.19*** | 0.144*** | 0.164*** | 0.183*** | 0.232*** | 0.115*** | 0.128*** | 0.078* | -0.024* | 0.188*** | 0.312*** | 0.297*** | 0.191*** | 0.296*** |
| CDC20 | 0.141*** | 0.205*** | 0.213*** | 0.188*** | 0.221*** | 0.2** | 0.098** | 0.149*** | 0.054* | 0.276*** | 0.378*** | 0.423*** | 0.122*** | 0.392*** |
| TTK | 0.184*** | 0.179*** | 0.175*** | 0.191*** | 0.245*** | 0.146*** | 0.142*** | 0.137*** | -0.003 | 0.204*** | 0.36*** | 0.336*** | 0.179*** | 0.345*** |
| NCAPG | 0.203*** | 0.163*** | 0.189*** | 0.198*** | 0.254*** | 0.16*** | 0.144*** | 0.093** | 0.005 | 0.228*** | 0.388*** | 0.362*** | 0.214*** | 0.367*** |
| LIPE | 0.097** | 0.017 | 0.023* | 0.062** | 0.01 | 0.037* | 0.138*** | 0.143*** | 0.188*** | -0.01 | 0.161*** | -0.221*** | -0.028* | -0.158*** |
| FABP4 | 0.14*** | 0.084** | 0.116*** | 0136*** | 0.091** | 0.118*** | 0.261*** | -0.14*** | 0.324*** | 0.027* | -0.051* | -0.16*** | 0.02 | -0.02 |

**D) Correlation analysis between related hub genes and gene markers of different T cell types in TIMER. (Th, T helper cell；Tfh, Follicular helper T cell;** **Treg, regulatory T cell)**

| Gene | Gene markers | | | | | | | | | | | | | |
| --- | --- | --- | --- | --- | --- | --- | --- | --- | --- | --- | --- | --- | --- | --- |
|  | Th1 | | | Th2 | | | Tfh | | | | Th17 | | Treg | |
|  | T-bet | STAT4 | IFN-r | GATA3 | STAT6 | IL13 | BCL6 | IL21 | CD278 | CXCL13 | STAT3 | IL17A | FOXP3 | CCR8 |
| ISG15 | 0.25*** | 0.157*** | 0.209*** | -0.024 | -0.061 | 0.11*** | -0.124*** | 0.126*** | 0.324*** | 0.171*** | 0.144*** | -0.044 | 0.28*** | 0.211*** |
| MX1 | 0.346*** | 0.153* | 0.315*** | 0.056 | 0.017 | 0.153*** | 0.009 | 0.206*** | 0.482*** | 0.234*** | 0.809*** | 0.081* | 0.412*** | 0.406*** |
| IFIT1 | 0.22*** | 0.268*** | 0.207*** | 0.14*** | 0.059 | 0.094** | 0.043* | 0.154*** | 0.378*** | 0.141*** | 0.795*** | 0.019 | 0.323*** | 0.378*** |
| DDX58 | 0.242*** | 0.347*** | 0.26*** | 0.193*** | 0.17*** | 0.079** | 0.127*** | 0.227*** | 0.41*** | 0.213*** | 0.524*** | 0.039* | 0.351*** | 0.44*** |
| STAT1 | 0.514*** | 0.546*** | 0.561*** | -0.049* | 0.06* | 0.19*** | 0.069* | 0.418*** | 0.713*** | 0.421*** | 0.513*** | 0.18*** | 0.635*** | 0.703*** |
| RSAD2 | 0.346*** | 0.408*** | 0.372*** | 0.12* | 0.028 | 0.423*** | 0.084* | 0.26*** | 0.561*** | 0.282* | 0.74*** | 0.111* | 0.116* | 0.271* |
| OAS1 | 0.27*** | 0.219* | 0.281*** | 0.18* | 0.055 | 0.311** | 0.035 | 0.213*** | 0.425*** | 0.266* | 0.71*** | 0.091* | 0.019 | 0.239* |
| IFI44 | 0.424*** | 0.161* | 0.409*** | 0.285* | 0.026 | 0.317** | 0.099* | 0.262*** | 0.575*** | 0.231* | 0.681*** | 0.052 | 0.029 | 0.17* |
| ITGAX | 0.516*** | 0.507*** | 0.437*** | -0.104** | 0.116** | 0.217*** | 0.184*** | 0.328*** | 0.564*** | 0.297*** | 0.159*** | 0.129*** | 0.573*** | 0.574*** |
| BUB1 | 0.226*** | 0.202*** | 0.331*** | 0.31*** | -0.153*** | 0.114*** | -0.122*** | 0.287*** | 0.439*** | 0.239*** | 0.114*** | 0.171*** | 0.392*** | 0.464*** |
| CCNB1 | 0.139*** | 0.108*** | 0.244*** | -0.214*** | -0.209*** | 0.084** | -0.158*** | 0.19*** | 0.342*** | 0.186*** | 0.158*** | 0.079* | 0.308*** | 0.353*** |
| BUB1B | 0.178*** | 0.175*** | 0.27*** | -0.224*** | -0.152*** | 0.1** | -0.114*** | 0.248*** | 0.38*** | 0.196*** | 0.058* | 0.155*** | 0.338*** | 0.436*** |
| CCNA2 | 0.227*** | 0.188*** | 0.333*** | -0.326*** | -0.217*** | 0.126*** | -0.14*** | 0.262*** | 0.434*** | 0.26*** | 0.116*** | 0.177*** | 0.373*** | 0.436*** |
| KIF11 | 0.171*** | 0.169*** | 0.268*** | -0.187*** | -0.178*** | 0.103** | -0.11*** | 0.23*** | 0.361*** | 0.201*** | 0.093** | 0.113*** | 0.333*** | 0.397*** |
| CDC20 | 0.227*** | 0.113*** | 0.293*** | -0.498*** | -0.305*** | 0.148*** | -0.23*** | 0.22*** | 0.364*** | 0.251*** | 0.236*** | 0.144*** | 0.336*** | 0.274*** |
| TTK | 0.188*** | 0.169*** | 0.311*** | -0.364*** | -0.243*** | 0.125*** | -0.173*** | 0.258*** | 0.404*** | 0.253*** | 0.063* | 0.167*** | 0.368*** | 0.41*** |
| NCAPG | 0.211*** | 0.187*** | 0.318*** | -0.294**8 | -0.262*** | 0.129*** | -0.144*** | 0.253*** | 0.421*** | 0.267*** | 0.131*** | 0.167*** | 0.368*** | 0.43*** |
| LIPE | 0.046* | 0.061* | 0.141*** | 0.346*** | 0.346*** | 0.029* | 0.21*** | -0.124** | 0.145*** | -0.154** | -0.152** | -0.143** | -0.076* | -0.078* |
| FABP4 | 0.111*** | 0.108*** | -0.076* | 0.162*** | 0.213*** | 0.041* | 0.132*** | -0.125** | -0.052* | -0.088** | -0.135** | -0.131** | -0.064* | -0.079* |
